# Assessing clinical utility of preconception expanded carrier screening regarding residual risk for neurodevelopmental disorders

**DOI:** 10.1038/s41525-022-00316-x

**Published:** 2022-07-29

**Authors:** Paranchai Boonsawat, Anselm H. C. Horn, Katharina Steindl, Alessandra Baumer, Pascal Joset, Dennis Kraemer, Angela Bahr, Ivan Ivanovski, Elena M. Cabello, Michael Papik, Markus Zweier, Beatrice Oneda, Pietro Sirleto, Tilo Burkhardt, Heinrich Sticht, Anita Rauch

**Affiliations:** 1 Institute of Medical Genetics, University of Zurich, Zurich, Switzerland; 2 Institute of Biochemistry, Friedrich-Alexander-Universität Erlangen-Nürnberg (FAU), Erlangen, Germany; 3 Medical Genetics, University Hospital Basel, Basel, Switzerland; 4 University Hospital Zurich, University of Zurich, Zurich, Switzerland; 5 University Children’s Hospital Zurich, University of Zurich, Zurich, Switzerland

**Keywords:** Preventive medicine, Genetic testing

## Abstract

The magnitude of clinical utility of preconception expanded carrier screening (ECS) concerning its potential to reduce the risk of affected offspring is unknown. Since neurodevelopmental disorders (NDDs) in their offspring is a major concern of parents-to-be, we addressed the question of residual risk by assessing the risk-reduction potential for NDDs in a retrospective study investigating ECS with different criteria for gene selection and definition of pathogenicity. We used exome sequencing data from 700 parents of children with NDDs and blindly screened for carrier-alleles in up to 3046 recessive/X-linked genes. Depending on variant pathogenicity thresholds and gene content, NDD-risk-reduction potential was up to 43.5% in consanguineous, and 5.1% in nonconsanguineous couples. The risk-reduction-potential was compromised by underestimation of pathogenicity of missense variants (false-negative-rate 4.6%), inherited copy-number variants and compound heterozygosity of one inherited and one *de novo* variant (0.9% each). Adherence to the ACMG recommendations of restricting ECS to high-frequency genes in nonconsanguineous couples would more than halve the detectable inherited NDD-risk. Thus, for optimized clinical utility of ECS, screening in recessive/X-linked genes regardless of their frequency (ACMG Tier-4) and sensible pathogenicity thresholds should be considered for all couples seeking ECS.

## Introduction

Targeted carrier screening in high-risk populations has been shown to reduce the incidence of several diseases, such as Tay-Sachs in Ashkenazi Jews, cystic fibrosis in White Europeans, and alpha/beta-thalassemia in Sardinians^1–3^. However, expanded carrier screening (ECS), where more genes or pathogenic variants are tested in a pan-ethnic manner, seems to be more cost-effective^4,5^. Additionally, clinical utility of preconception ECS has been shown regarding the reproductive decision making in couples found to be at-risk of having an affected child^6,7^, of which 62–77% pursued actions to avoid an affected pregnancy. Moreover, all of the 0.2–2.6% of couples found to be at-risk in other studies took action and opted for PGT-M (Preimplantation Genetic Testing for Monogenic disease)^8,9^. Nevertheless, the question of residual general risk after expanded carrier screening remains open and hampers genetic counselling.

Despite existing recommendations for gene panel design^10,11^, and variant pathogenicity classification^12^, the latter remains vague and most ECS studies differ greatly in numbers of genes tested ranging from tens to a few hundreds with little overlap^13–18^. Accordingly, the carrier and (estimated) at-risk-couple frequencies vary widely^13–18^.

The recently released ACMG guidelines provide criteria on gene selection for expanded carrier screening, but recommend replacing this term with “Tier 1–4 carrier screening”^19^. Tier-1 includes screening of *CFTR*, *SMN1* and medically and family-based risk genes, Tier 2 includes Tier-1 plus genes with carrier frequency ≥ 1/100, Tier-3 includes Tier-2 plus genes with carrier frequency ≥ 1/200 and X-linked conditions, and Tier-4 includes Tier-3 plus genes with carrier frequency < 1/200. The ACMG recommends to offer Tier-3 screening to all pregnant women and those planning a pregnancy, while for consanguineous pregnancies Tier-4 screening should be considered. Thereby, all tiers should be restricted to disease genes with at least moderately severe phenotypes.

Nevertheless, the recent ACMG guidelines do not recommend to provide a residual carrier-risk after ECS, because exact carrier frequencies and precise test sensitivities are not known^19^. Independent of the residual carrier-risk for specific genes, which may be reasonably well estimated^20^, the general question of how much of the reproductive risk can be detected by ECS remains vague. Since neurodevelopmental disorders (NDDs), especially intellectual disability (ID), in the offspring are a major concern of couples who wish to have children, we wondered how much of the risk for a child with disabling NDD (referring to ID, global developmental delay and autism spectrum disorder as defined by DSM-5)^21^ could be detected by ECS. The prevalence of ID is about 2–3% in Western countries, and 0.3–0.5% of the population fulfills the criteria of severe ID with IQs below 50^22^. ID has significant comorbidity with other NDDs and is most commonly accompanied by epilepsy, cerebral palsy, anxiety disorder, oppositional defiant disorder, and autistic disorder and many affected individuals have serious long-term health problems^23^. According to a large-scale exome sequencing study in NDD patients of European ancestry, 3.6% of cases are attributable to monogenic recessive coding variants, while 50% are explained by monogenic de novo coding variants^24^. In contrast, in NDD patients with consanguineous parents these figures are reversed with about 50% inherited recessive and 6% de novo likely causative coding variants^25,26^.

Previous ECS studies were commonly performed in general populations or fertility clinics in order to avoid bias through enrichment of disease risk. While this approach is reasonable to establish general carrier burden, it does not allow to address the question of residual NDD-risk, i.e. the remaining risk for a child with NDD after ECS, which may be due to limitations in variant detection and classification, gene content and non-inheritable or polygenic disease causes. Since determination of this risk in general populations would require huge cohort numbers and long-term follow-up data, we retrospectively studied the parental samples of a cohort of children with disabling NDDs, who previously had undergone extensive diagnostic work-up, in order to assess the sensitivity of ECS in detecting the NDD-risk in relation to various gene contents and variant classification approaches, as well as parental consanguinity status. In order to benchmark our findings with previous ECS studies, we also investigated monogenic recessive non-NDD genes.

## Results

### Comprehensive list of known autosomal recessive or X-linked disease genes

**Fig. 1:**
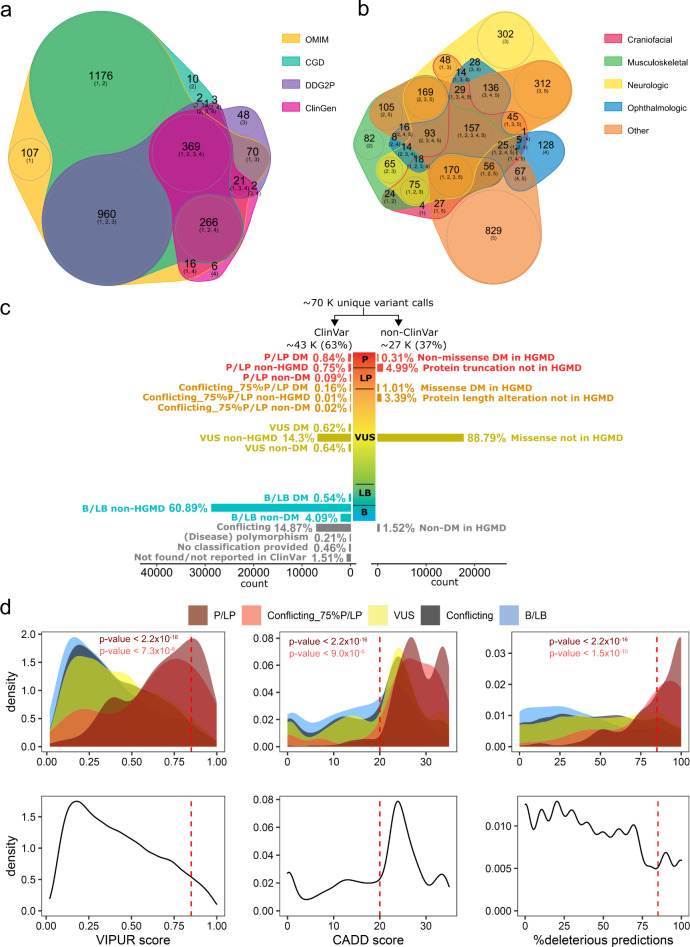
Genes and variant distributions. **a** Venn plot shows numbers of 3,058 AR/XL genes common or distinct in four databases, including the Online Mendelian Inheritance in Man (OMIM), the Clinical Genomic Database (CGD), the Development Disorder Genotype - Phenotype Database (DDG2P), and the Clinical Genome Resource (ClinGen). **b** Venn plot shows numbers of 3058 AR/XL genes in different manifestation categories according to the CGD manifestation category definition. **c** Bar plot shows distributions of the percentages of the filtered variants with (ClinVar) and without ClinVar annotation (non-ClinVar) according to their approximate levels of pathogenicity scale. P/LP: pathogenic/likely pathogenic; VUS: variant of uncertain significance; B/LB: benign/likely benign. **d** Density plots depict distributions of the filtered ClinVar pathogenic/likely pathogenic (P/LP, dark red), ClinVar conflicting with 75%P/LP entries (Conflicting_75%P/LP, red), ClinVar variants of uncertain significance (VUS, yellow), and ClinVar benign/likely benign (B/LB, light blue) missense variants according to VIPUR score (left), CADD score (middle), and the percentage of deleterious predictions of eight conventional in silico prediction tools, including SIFT, PolyPhen2, LRT, MutationTaster, MutationAccessor, FATHMM, PROVEAN, and M.CAP (right). P-values by Welch *t*-test show the significant difference of the distribution between P/LP and VUS/Conflicting/B/LB (dark red letters) and between Conflicting_75%P/LP and VUS/Conflicting/B/LB (red letters). Red dashed lines indicate cut-offs (VIPUR ≥ 0.85, CADD score ≥ 20, %deleterious predictions ≥ 85%) for “high stringency” missense variants (upper panel). Likewise, density plots depict distributions of the filtered non-ClinVar missense variants (lower panel).

We initially retrieved 3103 genes listed as AR or XL in at least one of the four databases OMIM, CDG, ClinGen, DDG2P. For final data evaluation we excluded 45 genes that were no longer considered recessive, and remained with 3058 genes, of which ~80% showed consensus recessive/X-linked inheritance (Fig. 1a and Supplementary Tables 1a, b, and 2). Although pLI-scores, which indicate the probability of intolerance to loss-of-function, may be used to discern between recessive and dominant genes with conflicting information^27^, we found that this parameter is generally not accurately categorizing well-established genes (Supplementary Table 1a). Clinical categorization revealed that 1990 of the remaining 3,058 genes affected neurological (1675) and/or musculoskeletal (1082) systems (Fig. 1b and Supplementary Table 1a). Four non-coding mRNA (*MIR2861*, *RMRP*, *RNU4ATAC*, *SNORD118*), one gastrointestinal (*PERCC1*), and three immunologic genes (*IGHM*, *IGKC*, *TRAC*) were not captured with standard exome kits. Since exome sequencing has limitations in detecting non-coding repeat-expansions or variants in genes that have a paralog or pseudogene, *FMR1*, *SMN1*, *GBA*, *HBA1, HBA2*, or *CYP21A2* could not reliably be assessed from the NGS data. Although *CYP21A2* is known to have a high carrier frequency in a middle European population^28^, it is not NDD-related, usually detected by biochemical new-born screening and well treatable. Since *SMN1* and *FMR1* represent the most common recessive severe muscle disorder and intellectual disability disease gene, respectively, we complemented the exome sequencing data of 3045 genes (including FMR1 sequence variants) by targeted testing for these two genes and thus analyzed a total of 3046 AR/XL genes, of which 1009 were annotated as definitive NDD genes in the sysNDD database, which includes genes causing developmental delay, intellectual disability and autism spectrum disorder. Despite improvement of gene capturing by newer exome kits, all methods applied in this study showed on average a 20-fold coverage of at least 90% of the coding region in more than 96% of all targeted recessive genes as well as of NDD genes (Supplementary Table 1a and Supplementary Fig. 1).

### Variant pathogenicity assessment

From the exome data of 3045 genes, we obtained ~70,000 different variants from 700, mostly European individuals (Supplementary Fig. 2), which we separated in ~43,000 (61%) ClinVar and ~27,000 (39%) non-ClinVar variants (Fig. 1c, Supplementary Fig. 3). Among the ClinVar variants, we found 791 P/LP, and another 86 variants with conflicting annotations with at least 75% of entries indicating P/LP. Of these, ~56% and ~95%, respectively, had entries in HGMD, with ~90% each classified as disease-causing mutation (DM) (Supplementary Fig. 3).

Using the ClinVar missense variants as benchmark dataset, the default VIPUR score cut-off of 0.5 to predict deleteriousness^29^ resulted in a specificity of 66% and a sensitivity of 78% (Fig. 1d). The support-vector-machine (SVM) model combining VIPUR and sequence-based prediction scores improved specificity to 99%, but limited sensitivity to 7%. Assessing various combinations of thresholds, we found that the cut-offs of VIPUR score ≥ 0.85, CADD score ≥ 20 and ≥ 85% of other sequence predictions being deleterious (Fig. 1d), yielded a 97% specificity and increased sensitivity to 24%. Applying these thresholds to our rare (minor allele frequency (MAF) < 2%) ~17,000 different non-ClinVar missense variants, which account for ~97% of the huge amount of VUS (Supplementary Fig. 3), we obtained 402 variants, referred to as non-ClinVar “high stringency”. Additionally, we found 1083 truncating and 346 in-frame non-ClinVar variants, of which 0.05% and 0.73% were HGMD-DM, respectively (Supplementary Fig. 3). From these 1831 non-ClinVar variants we excluded 44 with a gnomAD or internal MAF > 5% or > 2 homozygous/hemizygous alleles in gnomAD. After exclusion of variants in 45 genes that later were no longer considered recessive, we obtained for the 700 individuals 3674 variants in 3046 genes, which were classified into 16 variant classification groups according to evidence levels of pathogenicity (Supplementary Fig. 3).

Notably, gnomAD all population MAFs were absent or below 0.5% (majority below 0.1%) in all ClinVar P/LP variants in genes annotated to the SysNDD database as definitive NDD (Supplementary Fig. 4).

### Ethnical distribution and consanguinity in our cohort

Genetic ancestry was estimated using a projection Procrustes analysis tool, LASER, showing that 519 (74.1%) individuals clustered with the reference European populations; 117 (16.7%) and 54 (7.7%) clustered with the reference Central/South Asian and Middle East populations, and 10 (1.4%) clustered with other populations, respectively (Supplementary Fig. 2). Estimation by runs of homozygosity indicated in our cohort 23 (6.6%) consanguineous, 293 (83.7%) nonconsanguineous couples, and 34 (9.7%) couples with uncertain relationship (Supplementary Table 4a).

### Analysis of special disease alleles in *FMR1* and *SMN1*

We identified two mothers positive for an *FMR1* premutation (67 and 134 CGG repeats, respectively), indicating a female carrier frequency of 1/175. In only one of the latter the transmission of a full mutation to an affected boy explained his NDD, which was not diagnosed previous to this carrier screening study. Based on exome data we identified 36 potential heterozygous carriers for the recurrent *SMN1* ex7/8 deletion, of which 13 were confirmed by MLPA, accounting for a true carrier frequency of 1/54 despite any affected index cases (Supplementary Fig. 5).

### Final number of index cases with diagnoses

In addition to the 142 index cases initially diagnosed through an extensive diagnostic workup, five were diagnosed retrospectively through our carrier screening due to inclusion of novel disease genes (*MTX2*, *NARS1*, *NHLRC2*, and *TRAPPC4*), or *FMR1* screening (Supplementary Table 4b). The distribution of inheritance pattern and consanguinity of the total of 147 cases are detailed in Supplementary Table 4a.

### Overall carrier frequencies, at-risk couples and NDD risk-reduction potential

**Fig. 2:**
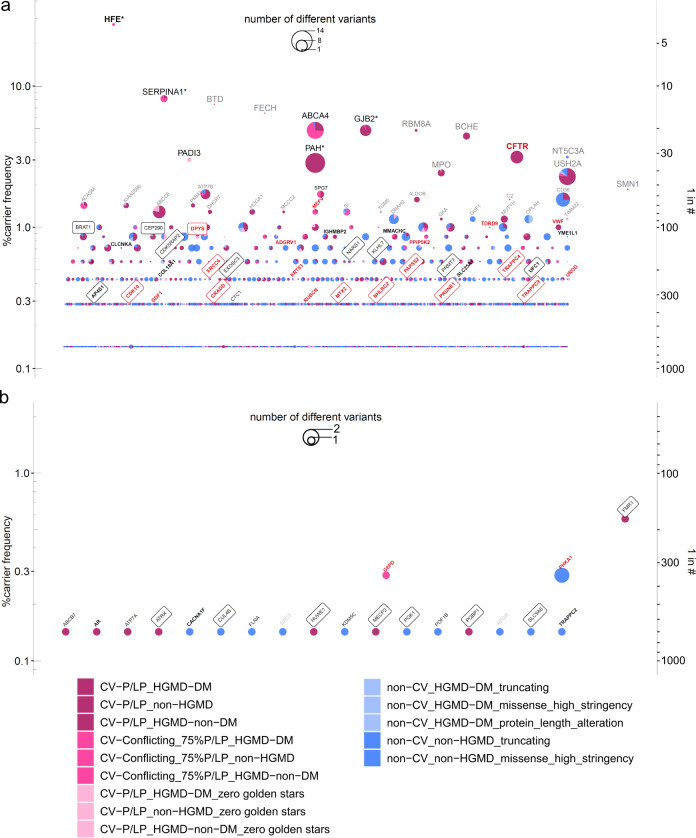
Carrier frequencies of recessive and X-linked genes observed in the 700 parental samples with indication of at-risk and transmitted at-risk genes and respective variant classification group distributions. Carrier frequencies of recessive (**a**) and X-linked genes (**b**). Carrier frequency was calculated as the percentage of the number of individuals carrying variants of different pathogenicity groups in each gene; on the right y-axis, a descriptive proportion was given. From top to bottom, the genes were sorted according to their carrier frequencies, as well as alphabetically wherever equal. Black letters indicate genes with at least one at-risk parental couple, red for genes with at least one at-risk consanguineous parental couple, bold for genes with at least one at-risk homozygous variant, and boxed for genes with at least one couple having transmitted both at-risk genotypes. Grey letters depict genes with > 1% carrier frequencies that were not found in an at-risk constellation. Stars indicate genes with more than one variant identified in at least one individual. Pie size correlates with the number of different variants in individual genes. The carrier frequencies were calculated among the 700 healthy individuals (350 parental couples) for 3046 recessive genes. The detected variants were shown for 14 variant classification groups according to their levels of pathogenicity, which include ClinVar pathogenic/likely pathogenic (P/LP) (CV-P/LP, in dark pink), ClinVar conflicting with 75% P/LP entries (CV-conflicting_75%P/LP, in pink), both stratified according to disease-causing mutation (DM) classification in the Human Gene Mutation Database (DM, non-HGMD, or HGMD-non-DM). The next groups include ClinVar variants with zero golden stars (in light pink), non-ClinVar with disease-causing mutation classification in the Human Gene Mutation Database (non-CV_HGMD-DM, in light blue) sub-categorized into variant functional classes including truncating, high stringency missense, or protein-length alteration, Non-CV_non-HGMD (blue) variants were sub-categorized according to variant functional classes including truncating, or high stringency missense.

**Fig. 3:**
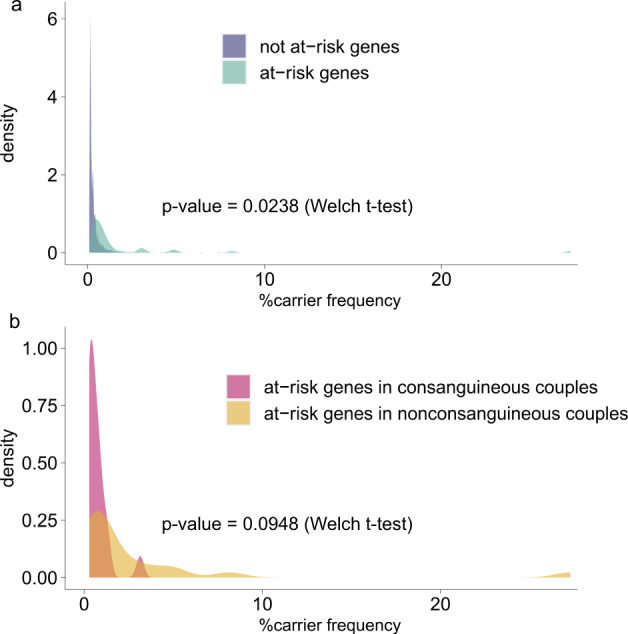
Effects of at-risk and consanguinity status on gene carrier frequencies. Considering variant classification groups 1–14, **a** Density plots show a significant difference between the mean of carrier frequency of genes that were at-risk and that of genes that were not at-risk according to their gene carrier frequencies. **b** Among the genes found in at-risk constellation, the mean of carrier frequency showed a trend towards lower frequencies in the consanguineous as compared to the nonconsanguineous group.

The distribution of clinical categorizations of genes showed neither a specific pattern in genes for which we found P/LP variants in individuals nor for genes with at-risk constellations in couples (Supplementary Fig. 6). Since variant classification groups 15 and 16 (non-ClinVar_non-HGMD non-canonical splice and protein length alterations) included many apparently benign variants, we did not consider them for further analysis. Accordingly, we found a total carrier frequency of up to 96.4% for at least one P/LP variant and a median of 4 P/LP variants per individual (range:0–12, Fig. 4, Supplementary Table 3a), which remained unchanged considering only autosomal genes (Supplementary Table 3b). While the majority of genes in which we found (likely) pathogenic variants showed frequencies ≤ 2%, 14 had frequencies > 2%, with *HFE* being most frequent (27.3%, Fig. 2). The mean of carrier frequencies for the definitive SysNDD genes was significantly lower than that for the other genes (0.28% vs. 0.40%, respectively, *p* = 0.007, Supplementary Table 1a), which is probably explained by natural selection against variants causing NDD. Considering established recessive SysNDD genes, top-ranked genes (frequency > 1%) included *PAH*, *KIAA0586*, *PMM2*, *DHCR7*, and *MCCC2* (Supplementary Table 1a), most of which exert biochemical phenotypes, with *PAH*, *PMM2* and *DHCR7* being treatable. We also observed that generally the mean of carrier frequencies of autosomal genes found in at-risk constellation was significantly higher than that of genes not found in at-risk constellation, which might be due to the inherent cohort bias (*p* = 0.0238) (Fig. 3a). However, within the genes found in at-risk constellation the mean of carrier frequencies showed a trend towards lower frequencies in consanguineous couples compared to nonconsanguineous couples (*p* = 0.0948) (Fig. 3b).

The real at-risk-couple frequency of 2.3–14.9% for autosomal recessive genes (3.1–19.1% with X-linked genes) was higher than those estimated by random virtual mating (0.5–9.4%), but became similar upon excluding recessive disease alleles found as diagnoses for the affected children (0.6–10.0%) (Supplementary Table 3a, b, Supplementary Fig. 7).

For autosomal recessive genes, up to 52 couples were at-risk for at least one gene. Of these couples, 21 (40.4%) harbored NDD at-risk genes, 27 (51.9%) non-NDD genes, and 4 (7.7%) both NDD and non-NDD genes (Supplementary Table 4b). Within these 52 at-risk couples (14.9% of all couples), consanguinity was enriched (13/52 = 25.0% vs. 10/272 = 3.7% in not-at-risk couples; *p* = 0.0001) (Supplementary Table 4b). Consanguinity was also significantly enriched in couples at-risk for > 1 autosomal gene (4/13 = 30.8% vs. 0/39 = 0%; *p* = 0.0026) (Supplementary Table 4b). 19 of the affected children inherited the at-risk alleles from the 25 at-risk NDD genes that explained their NDDs (Supplementary Table 4). This accounted for an autosomal risk-reduction potential for NDDs of 19/350 = 5.4%.

For X-linked genes, we identified up to 20 heterozygous female carriers equaling 5.7% at-risk couples. Of these, 13 (65%) concerned NDD genes, and 7 (35%) non-NDD genes. Eight of the 13 at-risk NDD alleles were transmitted to their children, including one girl who inherited a pathogenic variant in *MECP2* from her mother, corresponding to an X-linked risk-reduction potential of 2.3% (Supplementary Table 3c).

**Fig. 4:**
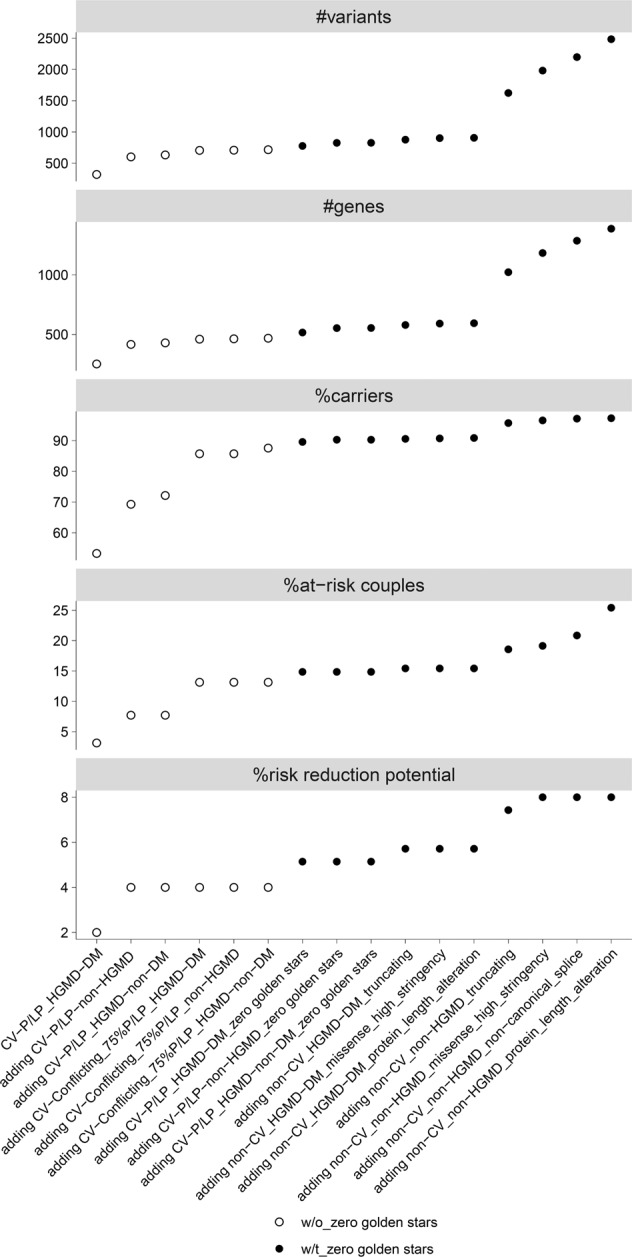
Summary of carrier testing. Results of carrier testing were summarized for number of detected variants, number of affected genes, and frequency of heterozygous carriers, at-risk couples, and risk-reduction potential according to the various pathogenicity thresholds, where a less conservative variant classification group is added stepwise as described for groups 1–14 in the legend of Fig. 2. It also includes the classification groups 15 (non-ClinVar_non-HGMD_non-canonical_splice) and 16 (non-ClinVar_non-HGMD_protein_length_alteration) which were not considered for further assessment. Overall, the carrier, and at-risk-couple frequencies showed the steepest increase upon inclusion of CV-P/LP_non-HGMD variants and the HGMD-DM, ClinVar conflicting variants with a high proportion of P/LP entries, as well as upon inclusion of previously unreported truncating variants. The risk-reduction potential showed a sharp increase upon inclusion of CV-P/LP_non-HGMD, CV-P/LP_HGMD-DM with zero golden stars, and non-CV_non-HGMD truncating variants.

Considering only the ClinVar and HGMD concordant P/LP variants for both AR and XL genes, the carrier and the at-risk-couple frequencies decreased by 44.8% and 83.6%, respectively (Fig. 4, Supplementary Table 3a). Additionally, removal of unreviewed ClinVar P/LP variants (zero golden stars) would decrease the risk-reduction potential by 14.3% from 7.7% to 6.6% through the exclusion of pathogenic variants in four genes (*CRADD*, *DPYS*, *TRAPPC9*, and *UFC1*) transmitted to the children of four couples (Supplementary Tables 4b, 5).

**Fig. 5:**
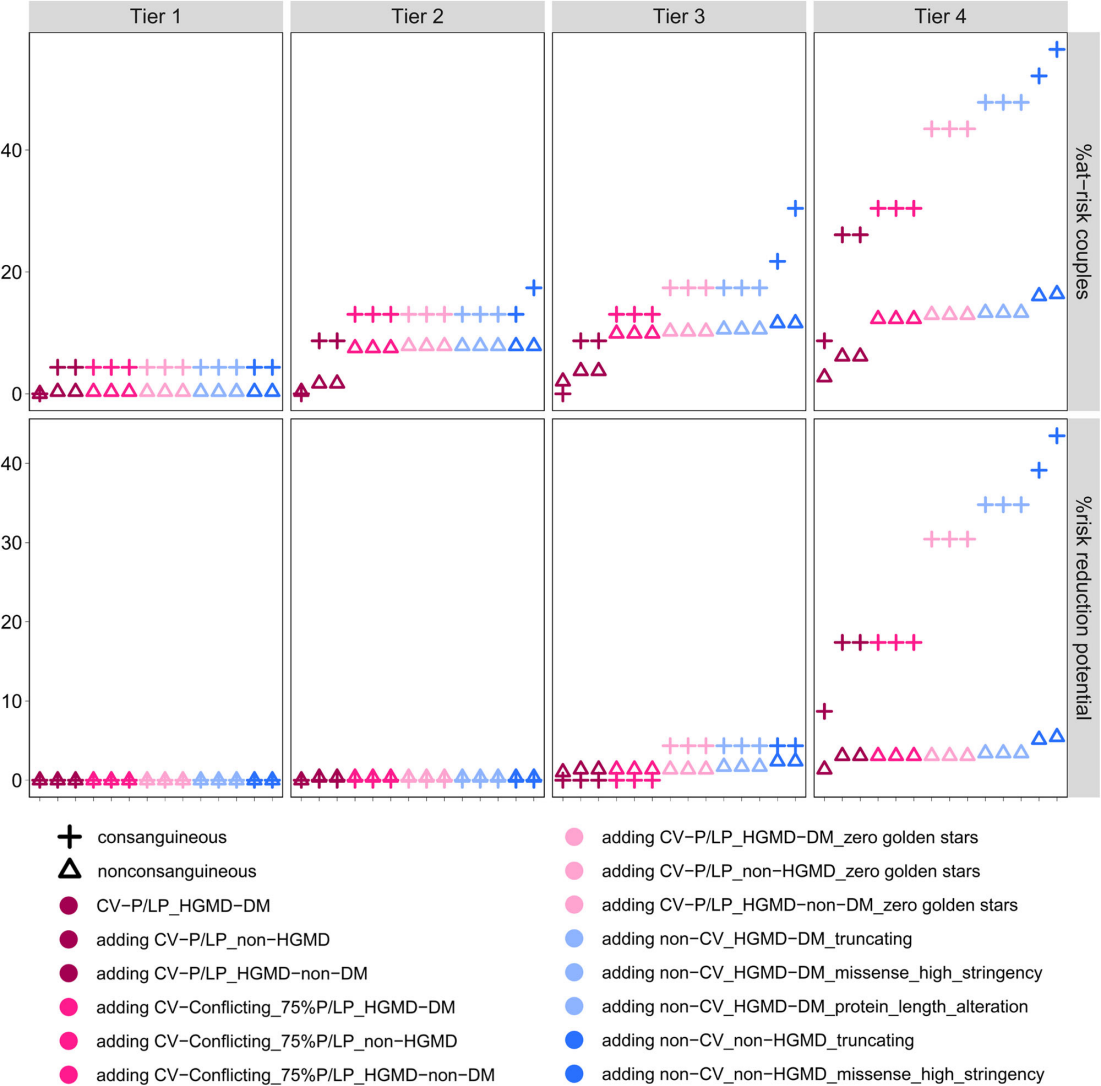
Magnitude of clinical utility of ACMG-based 4-Tier carrier screening. Frequency of at-risk couples and risk-reduction potential for NDDs according to a stepwise addition of the variant classification groups (1–14) are shown for nonconsanguineous and consanguineous couples for carrier screening for each of the 4-tiers recommended by the American College of Medical Genetics and Genomics (ACMG). In this carrier screening, Tier-1 includes screening of *CFTR*, *SMN1* and medically and family-based risk genes, Tier-2 includes Tier-1 plus genes with carrier frequency ≥ 1/100, Tier-3 includes Tier-2 plus genes with carrier frequency ≥ 1/200 as well as X-linked conditions, and Tier-4 includes Tier-3 plus genes with carrier frequency < 1/200.

Regarding the 4-Tier analysis scheme proposed by the ACMG committee for carrier screening^27^, we found that, based on carrier frequencies identified in our study, only when Tier-4 was applied, considerable increase of NDD risk-reduction potential not only for consanguineous (from none in Tier-2 to up to 43.5% in Tier-4, and up to ~10-fold from Tier-3 to Tier-4) but also for nonconsanguineous couples (from none in Tier-2 to up to 5.1% in Tier-4, and up to ~2.6-fold from Tier-3 to Tier-4) was achieved (Fig. 5). This also holds true for the at-risk-couple frequencies, in both consanguineous couples (up to ~3.2-fold increase by Tier-4 vs. Tier-2) and nonconsanguineous couples (up to ~1.5-fold increase by Tier-4 vs. Tier-2) (Fig. 5).

As expected, we found an enrichment of *de novo* pathogenic variants that explained the phenotype of the children in not-at-risk-couples compared to at-risk-couples (81/256 = 31.6% vs. 14/94 = 14.9%; *p* = 0.0017) (Supplementary Table 4b). There was no significant difference in paternal age between the *de novo* vs. inherited diagnoses (Supplementary Fig. 8). Finally, 203 (58.0%) couples remained without a diagnosis for their affected children, with no significant difference between the consanguineous and nonconsanguineous couples (12/23 = 52.2% vs. 172/293 = 58.7%, respectively; *p* = 0.66).

The NDD at-risk status of 16 (4.6%) couples remained undetected by ECS since the inherited causative variants of their affected children did not pass the filtering criteria (Supplementary Table 6). In addition, the risk for the recessive disorder in affected children was undetectable by ECS in 2 (0.6%) *de novo* hemizygous variants, 3 (0.9%) compound heterozygous inherited and *de novo* variants, and 3 (0.9%) inherited hemizygous CNVs or compound heterozygous inherited CNVs and sequence variants (Supplementary Table 4a).

## Discussion

We provide further evidence for clinical utility of ECS in prevention of NDDs by showing a high risk-reduction potential in consanguineous couples, albeit less so in nonconsanguineous couples. Our findings also revealed the magnitude by which the respective gene content and variant classification approaches influence the at-risk-couple detection rate and thus the risk-reduction potential.

### Variant classification

While it might seem straightforward to classify variants according to the ACMG guidelines, the latter are “intended for interpretation of variants observed in patients with suspected inherited (primarily Mendelian) disorders” and thus several criteria for pathogenicity assessment cannot be applied in the setting of ECS^12^. Therefore, theoretically, all rare variants that are not yet established as pathogenic could be considered as VUS. Nevertheless, truncating variants in genes where loss-of-function is a known mechanism of disease, have a very high probability of being pathogenic in recessive disorders, although rare exceptions exist^30^. Contrarily, missense and protein-length altering in-frame variants that are not already established as pathogenic will remain VUS since applicable ACMG criteria are usually limited to low population frequency and standard computational prediction tools. But even when only established pathogenic variants are considered, there is large room for interpretation what “established” means^31^. In terms of bioinformatic pipelines, interrogation of variant annotation in databases such as ClinVar or HGMD seems a feasible solution, but this concept is compromised by conflicting or insufficiently supported annotations. Since the DM (disease-causing mutation) classification by HGMD has been proven to be less specific for pathogenicity^32^, we prioritized ClinVar classifications for automatized annotation. Nevertheless, among previous studies there is no consent whether to consider variants with poor ClinVar review status or rare truncating variants without annotation. In addition, we observed that obvious P/LP variants may be labeled as “conflicting” due to few ClinVar entries classifying them as variant of unknown significance (VUS) or benign/likely benign (B/LB). While an automated ACMG variant classification tool such as InterVar may be applied for such variants^33^, this will likely underestimate pathogenicity due to lack of information about segregation and other non-automatable criteria. Among such ClinVar conflicting variants are the common hypomorphic variants in *HFE, SERPINA1, BTD*, and *FECH*, but also a relatively common *MMACHC* variant c.271_272insA with convincing evidence for pathogenicity (17/18 = 94% P/LP entries). Most previous carrier studies excluded such hypomorphic variants from their analysis^4,13,14,16,18,34^, while fewer did not^17,27,35^. Although we included these variants for our carrier frequency analysis, we did not consider them for at-risk couple analysis if not in a clinically relevant constellation with a pathogenic variant. Thus, the finding of only hypomorphic alleles in the same gene in both partners of 27 couples were not considered as at-risk status in our study, but may lead to overestimation of reproductive risk if included.

Given that rare missense variants without clinical annotation account for ~97% of the large amount of VUS (Supplementary Fig. 3), only one previous study evaluated such missense variants using CADD-scores^27^, which is however prone to false-positive classifications as we observed in our benchmarking of ClinVar annotated variants (Fig. 1d). Since this issue remained with structural evaluation using the VIPUR algorithm^29^, we finally applied a combination of VIPUR score ≥ 0.85, CADD score ≥ 20 and ≥ 85% of deleterious predictions from eight other predictive algorithms, which resulted in a high specificity (97%) albeit inevitably low sensitivity (24%).

### Carrier frequency, at-risk couples

In contrast to our study, which screened > 3000 AR and X-linked genes, ten previous studies interrogated < 500 genes^13–18,35–38^, one study screened 1,929 AR genes^27^, and one study sequenced 2350 AR genes in consanguineous couples, with variable P/LP definitions^34^. Our attempt to adjust our results according to the genes tested and P/LP definitions applied by each of the previous studies revealed mostly higher carrier and at-risk frequencies, which could be attributable to the likely bias for NDD carrierships in our cohort (Supplementary Fig. 9). However, in general these carrier frequencies exhibited asymptotic increases with increasing numbers of genes tested, which tends to saturate at higher gene numbers (Supplementary Fig. 9). This finding agrees with a previous observation, where the cumulative carrier frequency asymptotically increased with increasing gene number^13^. Contrarily, the at-risk-couple frequencies seem to increase linearly with increasing gene numbers, especially for variants with higher evidence level for pathogenicity (Supplementary Fig. 9).

Given the previous prediction that if the yet undiscovered estimated 2000–6000 AR genes are to be included, the median carrier frequency should be 4–5 P/LP variants per individual^27^, seems to underestimate the actual range, given that we already found a median of 4 P/LP variants per individual for 3046 genes (even when removing frequent hypomorphic alleles and disease alleles of the affected children). Moreover, neither our study nor the other studies investigating larger gene sets evaluated copy-number variants from the NGS data in general, which would likely increase the carrier frequency even more.

Despite the lack of affected children with spinal muscular atrophy in our cohort, we identified an *SMN1* carrier frequency of 1/54, which is in the higher range reported for different populations (1/100–1/50) and in line with the expectation for a mainly European cohort^14,16,34,39^. In contrast, the *FMR1* carrier frequency of 1/175 in women of our cohort is in the lower range compared to reported frequencies (1/169–1/80)^14,16^, although one patient was diagnosed with fragile X syndrome following the result of our carrier screening.

Despite all the discrepancies, previous studies that analyzed real couples reported relatively higher at-risk-couple frequencies (0.6–4.4%)^14,16,17,35,39^ than those that simulated virtual couples of random mating (0.17–2.52%)^4,13,18^. Among the former, the studies that included panethnic populations showed at-risk-couple frequencies in the lower range (0.6–3.6%)^16,17,39^, while those that contained a majority of one specific population exhibited at-risk-couple frequencies in the higher range (4.35–4.40%)^14,35^, probably due to non-random mating or cohort bias. In line with this assumption, the observed at-risk-couple frequency in our cohort is similar to that estimated by random mating after excluding the disease alleles of the affected children.

According to the “Mackenzie’s Mission project”, in 2020 there were 1300 recessive genes associated with a severe phenotype such that couples would be likely taking steps to avoid an affected child^40^. Adjusting our results to these genes, the maximum carrier frequency would drop to 84.7% with a median of two P/LP variants per individual and real at-risk-couple frequency of up to 10.3%. Notably, the estimated at-risk-couple frequency with random “mating” of our cohort for these 1,300 genes would be only 0.3–1.7%, which is comparable to the frequency of 0.9% estimated in a recent study analysing 1119 Mackenzie’s severe genes^27^. Importantly, these 1300 severe genes did not contain eight of the genes diagnosed in the index children, thereby screening only such outdated gene-lists would have missed ~30% of our recessive diagnoses (Supplementary Table 4b).

Notably, although consanguinity did not affect the number of P/LP autosomal variants per individual in our cohort, it significantly increased the at-risk-couple frequency, i.e., from up to 48/293 = 16.4% in nonconsanguineous couples to up to 13/23 = 56.5% in consanguineous couples (*p* = 0.0001). In line with this, a recent carrier study on 100 consanguineous couples that screened 2350 autosomal recessive genes identified an at-risk-couple frequency of 58%^34^. Enrichment of consanguinity among at-risk couples is in line with previous observations that consanguinity is a risk factor that facilitates transmission of at-risk genotypes^41^. Of note, while our data in general support the hypothesis that the at-risk frequency of a gene is increased with increasing variant frequency in a given population^13^, for consanguineous couples the genes that were found in an at-risk constellation are more likely to have a low variant frequency (Fig. 3).

### Risk-reduction potential for NDDs

We found that the risk for the NDD diagnosed in the respective affected child was detected by our carrier screening in up to 7.7% (27 of 350 affected children) when including the first 14 variant classification groups with a decreasing rate for more conservative approaches (Fig. 4, Supplementary Table 3a). The biggest leap in values occurs upon inclusion of ClinVar-P/LP_HGMD-DM zero golden stars variants in consanguineous couples and non-ClinVar_non-HGMD truncating variants in nonconsanguineous couples, respectively (Fig. 5). Notably, this risk-reduction potential was much higher in consanguineous couples (up to 43.5%) than in nonconsanguineous couples (up to 5.1%) (Fig. 5), which is in line with the known risk of autosomal recessive disorders in offspring of consanguineous couples^41^. Nevertheless, despite the ACMG recommendation to consider Tier-4 carrier screening only for consanguineous matings^27^, our data indicate, that in nonconsanguineous couples without Tier-4 screening > 50% risk-reduction potential would be missed (2.0% instead of 5.1%). Therefore, concerning clinical utility, Tier-4 screening should be considered regardless of consanguinity status. However, given that in nonconsanguineous couples 94.9% of NDD children could not be anticipated, performing an ECS in such couples may require a disclaimer for limited utility.

Of note, the total risk-reduction potential would have been increased by 60% (from 7.7% to 12.3%) if not 17 missense variants would have been filtered out due to stringency reasons (Supplementary Table 6), which in the clinical setting were categorized as likely pathogenic considering non-automatable parameters such as phenotype, segregation and in-depth variant assessment. Therefore, sensitivity of our ECS approach for detectable inherited risk was up to 62.8% (27/43). Notably, a stringent cut-off of 8 alleles in gnomAD as recommended by Sherloc, et al.^42^ would filter out one high stringency missense variant and reduce the risk-reduction potential to 7.4% and sensitivity to 60.5% (26/43) (couple ID 174, Supplementary Table 4). Since attempts to increase sensitivity while retaining specificity for variant classification by in silico predictions remained unsatisfactory, a timely submission of such clinically assessed likely pathogenic variants into a disease variant database such as ClinVar is warranted to increase the sensitivity of carrier screening. Likewise, a continuous update of genes analysed in carrier screening is mandatory to harvest the full clinical utility of Tier-4 carrier screening in the light of the still growing number of recessive disease genes.

Notably, 0.9% of NDD-patients had inherited pathogenic CNVs as a disease-cause (hemizygous or compound heterozygous) indicating ~1% higher risk-reduction potential in carrier screening when including CNV analysis. Another 0.9% of patients had an AR disorder caused by one inherited and one *de novo* pathogenic variant, a possibility that is commonly neglected in genetic counselling. Moreover, a considerable number of couples had their children diagnosed with a dominant (27.1%) or mitochondrial (0.3%) disorder caused by *de novo* pathogenic variants, which was enriched among the certain nonconsanguineous couples with diagnoses in their children (87/121 = 71.9% vs. 1/11 = 9.1% among the certain consanguineous couples, *p* = 0.0001). This agrees with the finding from the Deciphering Developmental Disorders study, where *de novo* dominant pathogenic variants account for the majority of the diagnostic yield in outbred populations^43^.

Considering the impact on at-risk couple frequency and risk reduction potential demonstrated above, all recessive/X-linked genes, regardless of their carrier frequencies (if existing) should be considered in ECS independent of parental consanguinity status. In order to achieve the highest sensitivity without compromising specificity, gene lists and ClinVar annotations should be updated regularly, and pathogenicity of the obtained variants should be assumed for variants annotated in ClinVar as P/LP with at least one golden star. ClinVar variants with P/LP zero golden star or conflicting but mainly P/LP annotation should be assessed manually, which is feasible due to their rare average cumulative occurrence of less than 0.5 per sample. Non-ClinVar truncating variants contributed considerably to the risk reduction potential and should be considered likely pathogenic if they are not found in population databases or if their minor allele frequency is below 0.5%. In case one of the partners harbours variants fulfilling one of the above criteria, the respective genes should also be assessed for non-ClinVar missense and protein-length altering variants with MAFs below 0.5% in the other partner. Such variants may be further evaluated for entries in HGMD and other disease databases, as well as by a variety of prediction tools. By this approach at-risk couple frequencies would slightly drop from 16.4% to 16.0% in nonconsanguineous couples and from 56.5% to 52.2% in consanguineous couples without requiring extensive pathogenicity modelling in missense variants with its inherent risk for compromised specificity.

In conclusion, our findings revealing the magnitude of utility of preconception ECS for NDD-risk in relation to parental consanguinity, screened gene content and pathogenicity assessment will inform genetic counselling in reproductive medicine. To further increase utility of carrier screening, improved variant classification and identification of novel disease genes are warranted.

## Methods

### Exome sequencing and cohort characteristics

Written informed consent was obtained from all human participants. Trio-exome sequencing had been performed for 350 children with NDDs and their parents on peripheral blood DNA using Agilent SureSelect XT Clinical Research Exome Kit (V5), or Human All Exon (V6), or Twist Human Core Exome Kit (Twist Bioscience), on a HiSeq 2500 System (Illumina, CA, USA) with 125-bp paired-end reads as described elsewhere^44^, or xGen Exome Research Panel (IDT v1.0 or IDT v2.0) on a NovaSeq 6000 System (Illumina Inc.) with 150-bp paired-end reads. Sequence alignment and variant calling was performed using NextGene V2.4.2.3 (Softgenetics). For usage of the Automap software^45^, data were realigned with an Illumina DRAGEN Bio-IT Platform (version 3.7.5). Analysis for pathogenic or likely pathogenic (P/LP) variants in the children had been performed as part of research studies investigating the genetic landscape of NDDs, parts of which have been published^46,47^. 350 parental couples (designated “p001–p350”, with appending “f” or “m” for female or male, respectively; their affected children as “c001–c350”) had consented to further studies and/or investigation of secondary findings; thus, their data were used for carrier screening. The median age at birth of the affected child was 31.2 (range: 17.3–44.0) years for the mothers and 34.5 (range: 18.8–64.3) for the fathers. Of the 350 affected children, 40.6% (142) had genetic diagnoses prior to carrier screening in their parents. Of these, 62.0% (88/142) had causative dominant *de novo* sequence variants (SV), 17.6% (25/142) autosomal recessive inherited SVs, 8.5% (12/142) X-linked recessive inherited SVs, 4.9% (7/142) dominant *de novo* copy-number variants (CNVs), 1.4% (2/142) X-linked recessive inherited CNVs, 1.4% (2/142) X-linked recessive *de novo* SVs, 0.7% (1/142) X-linked dominant maternally inherited SV, 0.7% (1/143) autosomal dominant maternally inherited SV, 2.1% (3/142) compound heterozygous inherited and *de novo* SVs, 0.7% (1/142) compound heterozygous inherited SV and CNV (Supplementary Table 4a).

This study was approved by the cantonal ethics committee of Zurich, references BASEC-Nr. PB_2016-02520 and 2019-00016.

### Selection and categorization of recessive genes

Known genes annotated with autosomal recessive or X-linked inheritance for a monogenic disorder were selected from four disease gene databases (the Online Mendelian Inheritance in Man (OMIM, https://www.omim.org/): genemap2.txt, downloaded 26 March 2021, the Clinical Genomic Database (CGD, https://research.nhgri.nih.gov/CGD/): CGD.txt, downloaded 30 March 2021, the Development Disorder Genotype - Phenotype Database (DDG2P, https://www.deciphergenomics.org/ddd/ddgenes): DDG2P_07_04_2021.csv, downloaded 7 April 2021, and the Clinical Genome Resource (ClinGen, https://clinicalgenome.org/): Clingen-Gene-Disease-Summary-2021-04-08.csv, downloaded 8 April 2021) (Supplementary Table 1a). Consensus inheritance of autosomal genes was determined for genes annotated in more than one database. Genes described for autosomal recessive inheritance in each database were considered consensus, and otherwise as conflicting. Genes annotated as autosomal dominant, only, in more than one database, i.e. CGD and ClinGen, were manually curated in the literature as to whether they are also described for autosomal recessive inheritance (Supplementary Table 2). Genes that became annotated as autosomal dominant in OMIM during the course of the study were retained if annotated as recessive/X-linked in the SysNDD database, which encompasses developmental delay (DD), intellectual disability (ID) and autism spectrum disorder (ASD) (https://sysndd.dbmr.unibe.ch/), or if manually curated as also being recessive (Supplementary Table 1a). All X-linked genes associated with a monogenic disorder were considered consensus X-linked inheritance. Gene categories were mostly based on the clinical manifestation categories from the CGD database. Four autosomal recessive RNA genes, *MIR2861*, *RMRP*, *RNU4ATAC*, and *SNORD118*, three immunologic genes, *IGHM*, *IGKC*, and *TRAC*, and one gastrointestinal gene *PERCC1* were excluded from our analysis due to the capturing limitation of the used standard exome capture kits (Agilent SureSelect V5/V6, TWIST and xGen IDT v1/v2). One gene, *TUBB8*, was not present in the oldest kit (SureSelect V5), but no rare variants were detected in this gene in any of the kits used.

### Variant filtering and classification for carrier analysis

Variants with overall quality score ≥ 12 were obtained (Supplementary Fig. 3). All variants present in the ClinVar database were annotated for their ClinVar classification (https://www.ncbi.nlm.nih.gov/clinvar/, updated per 28 March 2021). Variants not annotated in ClinVar that were in the untranslated region, farther than three intronic bp, and synonymous, were excluded. For both ClinVar and non-ClinVar variants, variants observed in < 28% alternative reads in the proband as well as in multiple probands with an average of < 28% alternative reads in at least 90% of these probands were discarded as likely artefacts. Variants within homology regions^48^ or exhibiting an extreme forward-to-reverse read ratio (< 0.2 or > 0.8) were also excluded. Remaining variants were also annotated according to their pathogenicity classifications in the Human Gene Mutation Database (HGMD Professional version 2021; https://my.qiagendigitalinsights.com/bbp/view/hgmd/pro/start.php: batch searched 24 April 2021). Variants were categorized into pathogenicity threshold groups, based on their classifications in ClinVar and HGMD database, and variant functional classes, including truncating, non-canonical splice, protein length alteration, and missense (Supplementary Fig. 3). The non-ClinVar variants with an overall minor allele frequency > 5%, a cohort-specific allele frequency > 5%, or with > 2 homozygotes/hemizygotes reported in the gnomAD database (https://gnomad.broadinstitute.org/) were excluded. Variants in genes with carrier frequency > 2% in our cohort were manually curated to exclude likely polymorphisms or dominant alleles before calculating the final carrier frequencies.

### Computational classification of missense variants

Pathogenicity of missense variants was analyzed using eight sequence-based predictions (SIFT, PolyPhen2, LRT, MutationTaster, MutationAccessor, FATHMM, PROVEAN, M.CAP), CADD score, (embedded in NextGene V2.4.2.3), and automated assessment of structural effects using VIPUR (Variant Interpretation and Prediction Using Rosetta) (accessed at https://osf.io/bd2h4/ on 28 March 2018), which was reported to exhibit a good performance in distinguishing between neutral and deleterious protein variants^29^. In order to generate a large dataset of structures for variant modeling, VIPUR used two major resources of protein models, namely SwissProt^49^ (accessed at https://swissmodel.expasy.org/repository on 2 April 2020) and ModBase^50^, (accessed at https://salilab.org/modbase-download/ on 9 November 2019) which were combined into a common database containing 513,000 structural models. To save computer time, for large protein structures only the domain harboring the variant of interest was included in the VIPUR analysis. Domains were selected based on domain detection^51^ or PFAM^52^ (accessed at http://hmmer.org on 30 March 2020) domain annotation, respectively; in case of a missing PFAM annotation, sequence domains of 400 residues (centered around the site of variants) were used for analysis. In total, 33,971 variants, which included non-B/LB (before update per 28 March 2021) ClinVar and non-ClinVar (MAF ≤ 2%) missense, were classified by VIPUR. A Support Vector Machine (SVM) model using an R package “e1071” was used to predict pathogenicity for the non-ClinVar missense variants based on the VIPUR scores, the eight sequence-based scores, and CADD scores, to filter for “high stringency” missense variants.

### *SMN1* copy number detection

*SMN1* copy numbers were calculated based on an approach previously described^53^. Briefly, raw proportions of *SMN1* reads over the total number of *SMN1* and *SMN2* reads were calculated at three genomic positions that differ between the two genes, *SMN1*: chr5:70247724, chr5:70247773, chr5:70247921, and *SMN2*: chr5:69372304, chr5:69372353, chr5:69372501. The raw proportions were scaled with a scale factor, which was derived by normalizing the total number of *SMN1* and *SMN2* reads for a set of 20 control genes within samples in the same run. Scaled proportions less than 1/3 were considered potential carriers. The scale factor and the raw proportion of *SMN1*/*SMN2* reads at the three gene-specific positions were used to estimate the absolute copy number, as well as to cluster potential carriers by a principal component analysis. Potential carriers were validated by MLPA targeting dosage of exons 7 and 8 of the *SMN1* and *SMN2* genes using the kit P060, vs B2-04 lot B2-0218 (MRC, Holland). The products were analyzed using an ABI3730 and the Coffalyser Software (MRC, Holland). Reference sequences: *SMN1*: NM_000344.3 (LRG_676t1); *SMN2*: NM_017411.3.

### *FMR1* testing

The analysis of the *FMR1* alleles with CGG repeats in the normal range up to full mutations (normal range: up to 45 CGG repeats; intermediate alleles: 45-54 repeats; premutation range: 55 to 200 repeats; full mutation: more than 200 repeats)^54^ was performed using the RP PCR analysis Kit (AmplideX, lot: 18761, Asuragen) in all available mothers. *FMR1* reference sequence: LRG_762t1.

### Calculation of carrier frequency, at-risk couples, and risk-reduction potential

Carrier frequency for a given gene was calculated as the percentage of the number of individuals carrying at least one pathogenic variant in that gene among the total number of individuals investigated. Total carrier frequency was calculated as followed:

$${{{\mathrm{total}}}}\,{{{\mathrm{carrier}}}}\,{{{\mathrm{frequency}}}} = \frac{1}{{N_{{{{\mathrm{total}}}}\,{{{\mathrm{individuals}}}}}}} \cdot \mathop {\sum}\limits_{i = 1}^{n_{{{{\mathrm{genes}}}}}} {\left( {N_{{{{\mathrm{carriers}}}}\,{{{\mathrm{of}}}}\,{{{\mathrm{gene}}}}\,i}} \right)}$$

Actual parental couples were at-risk if they each have at least one (likely) pathogenic variant in the same gene. “At-risk” constellations of two hypomorphic variants that are known to be clinically not relevant were excluded. Variants contributing to parental at-risk constellations were visually examined using NextGene Viewer software to remove possible artifacts. Estimated at-risk couple rates were based on actual genotypes and identified as followed:

$$\frac{{\mathop {\sum}\nolimits_{i = 1}^N {\left( {\begin{array}{*{20}{c}} {number\,of\,carriers\,of\,gene\,i} \\ 2 \end{array}} \right)} }}{{\left( {\begin{array}{*{20}{c}} {total\,number\,of\,individuals} \\ 2 \end{array}} \right)}},{{{\mathrm{for}}}}\,{{{\mathrm{AR}}}}\,{{{\mathrm{genes}}}};$$

$$\frac{{\mathop {\sum}\nolimits_{i = 1}^N {\left( {\begin{array}{*{20}{c}} {number\,of\,carriers\,of\,gene\,i} \\ 1 \end{array}} \right)} }}{{\left( {\begin{array}{*{20}{c}} {total\,number\,of\,individuals} \\ 2 \end{array}} \right)}},{{{\mathrm{for}}}}\,{{{\mathrm{XL}}}}\,{{{\mathrm{genes}}}}.$$

Autosomal genes for which both partners of a couple are heterozygous carriers of a P/LP variant and X-linked genes for which the female partner is heterozygous carrier of a P/LP variant were referred to as “at-risk genes” and the respective variants as “at-risk variants”. Risk-reduction potential was calculated as the percentage of couples in which diagnoses of the affected offspring matched the at-risk genotypes.

### Estimation of degree of relationship by runs of homozygosity

Runs of homozygosity (ROH) were analzed using AutoMap version 1.0^45^. Degrees of consanguinity were estimated based on inbreeding coefficient (F_ROH_), where first, second, or third degree of relationship was considered consanguinity^55,56^. F_ROH_ was derived as a proportion of total autosomal ROH > 5 Mb ($L_{ROH\,auto}$) (a threshold likely representing identity by descent)^57^ to the total autosomal genomic length ($L_{auto}$, approximately 2691 Mb for GRCh37/hg19)^56^, as followed:

$$F_{ROH} = \frac{{{\sum} {L_{ROH\,auto}} }}{{L_{auto}}}$$

### Genetic ancestry estimation

Genetic ancestry was estimated using a projection Procrustes analysis tool, LASER version 2.04 (Locating Ancestry from SEquence Reads) (https://laser.sph.umich.edu/index.html#!)^58^. Briefly, raw BAM files were converted to more compact LASER’s native sequence format, and loci with less than 20 reads in more than 10% of individuals were removed, resulting in 16,039 loci. These loci were subjected to the LASER tool against the same loci from the reference populations from the Human Genome Diversity Project (HGDP), which were available in the LASER tool. The three principal components from LASER were used for a cluster analysis by the k-nearest neighbors algorithm among the reference samples to estimate ancestry (Supplementary Fig. 2).

### Statistical analysis

Significance was evaluated with Welch’s *t*-test, or two-tailed Fisher’s exact test for comparison of proportions using R scripts. Results with *p*-value ≤ 0.05 were considered statistically significant.

### Reporting summary

Further information on research design is available in the Nature Research Reporting Summary linked to this article.

## Supplementary information

Reporting Summary

Supplementary Information

Dataset 1

## Data Availability

Data that support the findings of this study are available in the supplementary tables. Due to the limitations of the ethics approval and informed consent signed by the participants, the raw exome sequencing data used for this study cannot be deposited into a public repository; however, ethical approval and consent does allow for data sharing but only in the context of an approved study collaboration. These raw data are available from the SwissGenVar database (https://sphn.ch/network/projects/infrastructure-development-projects/project-page-swissgenvar/). Requests for access to these data must comply consortium rules and require a Data Transfer and Use Agreement, as well as ethical approval for the usage of these data. Correspondence and requests for access to these data should be addressed to swissgenvar@medgen.uzh.ch.

## Code availability

NextGene V2.4.2.3 (Softgenetics Inc.), Automap v1.0, SwissProt (accessed at https://swissmodel.expasy.org/repository on 2 April 2020), ModBase (accessed at https://salilab.org/modbase-download/ on 9 November 2019), PFAM (accessed at http://hmmer.org on 30 March 2020), LASER version 2.04 (https://laser.sph.umich.edu/index.html#!), and R version 4.1.0, ClinVar (https://www.ncbi.nlm.nih.gov/clinvar/, 28 March 2021), HGMD Professional 2021 (Qiagen Inc.), VIPUR (accessed at https://osf.io/bd2h4/ on 28 March 2018); SIFT, PolyPhen2, LRT, MutationTaster, MutationAccessor, FATHMM, PROVEAN, M.CAP, and CADD score (embedded in NextGene V2.4.2.3). Variant filtering was conducted using custom code in R (4.1.0), which is available upon reasonable request.
