# Supplementary material for: Assessing clinical utility of preconception expanded carrier screening regarding residual risk for neurodevelopmental disorders
Source: NPJ Genom Med. 2022 Jul 29;7:45. doi: 10.1038/s41525-022-00316-x (PMC9338263; doi:10.1038/s41525-022-00316-x)
Supplement: Supplementary file 2 — Supplementary Information [file 41525_2022_316_MOESM2_ESM.pdf]

Supplementary Information for

Assessing clinical utility of preconception expanded carrier screening regarding residual risk for neurodevelopmental disorders

Paranchai Boonsawat, PhD,[1] Anselm HC Horn, PhD,[1,3] Katharina Steindl, MD,[1] Alessandra Baumer, PhD,[1] Pascal Joset, PhD,[2] Dennis Kraemer, MSc,[1] Angela Bahr, PhD,[1] Ivan Ivanovski, MD,[1] Elena M Cabello, MSc,[1] Michael Papik, MSc,[1] Markus Zweier, PhD,[1] Beatrice Oneda, PhD,[1] Pietro Sirleto, MSc,[1] Tilo Burkhardt, MD,[4] Heinrich Sticht, PhD,[3] Anita Rauch, MD[1,5]

[1]Institute of Medical Genetics, University of Zurich, Zurich, Switzerland

[2]Medical Genetics, University Hospital Basel

[3]Institute of Biochemistry, Friedrich-Alexander-Universität Erlangen-Nürnberg (FAU), Germany

[4]University Hospital Zurich, University of Zurich, Zurich, Switzerland

[5]University Children's Hospital Zurich, University of Zurich, Zurich, Switzerland

Correspondence: Prof A Rauch MD (anita.rauch@medgen.uzh.ch)

Supplementary appendix

Table of contents

Supplementary figures ... 2

Supplementary Figure 1: 20x exome coverage of genes tested in our study ... 2

Supplementary Figure 2: Ancestry estimation by LASER (Locating Ancestry from SEquence Reads) analysis ... 3

Supplementary Figure 3: Variant filtering ... 5

Supplementary Figure 4: Distribution of all population minor allele frequencies of ClinVar P/LP variants in SysNDD genes ... 6

Supplementary Figure 5: Principal component analysis for the identification of potential *SMN1* carriers from exome sequencing data. ... 7

Supplementary Figure 6: Distribution of genes in which P/LP variants were detected with reference to manifestation categories. ... 8

Supplementary Figure 7: Actual versus estimated at-risk couple frequencies ... 9

Supplementary Figure 8: Distribution of paternal age in children with causal *de novo* and inherited pathogenic variants, respectively ... 10

Supplementary Figure 9: Comparison of carrier and at-risk rates between carrier studies. ... 11

Supplementary tables ... 12

Supplementary Table 2: Literature search for the genes with autosomal dominant inheritance in DDG2P and ClinGen ... 12

Supplementary Table 3a: Summary of carrier analysis results for autosomal recessive and X-linked genes according to increasing levels of variant pathogenicity thresholds ... 13

Supplementary Table 3b: Summary of carrier analysis results for autosomal recessive genes according to increasing levels of variant pathogenicity thresholds ... 14

Supplementary Table 3c: Summary of carrier analysis results for X-linked genes according to increasing levels of variant pathogenicity thresholds ... 15

Supplementary Table 6: Couples with recessive genetic diagnoses in their affected child not passing the stringent filtering criteria in carrier-screening ... 16

Supplementary figures

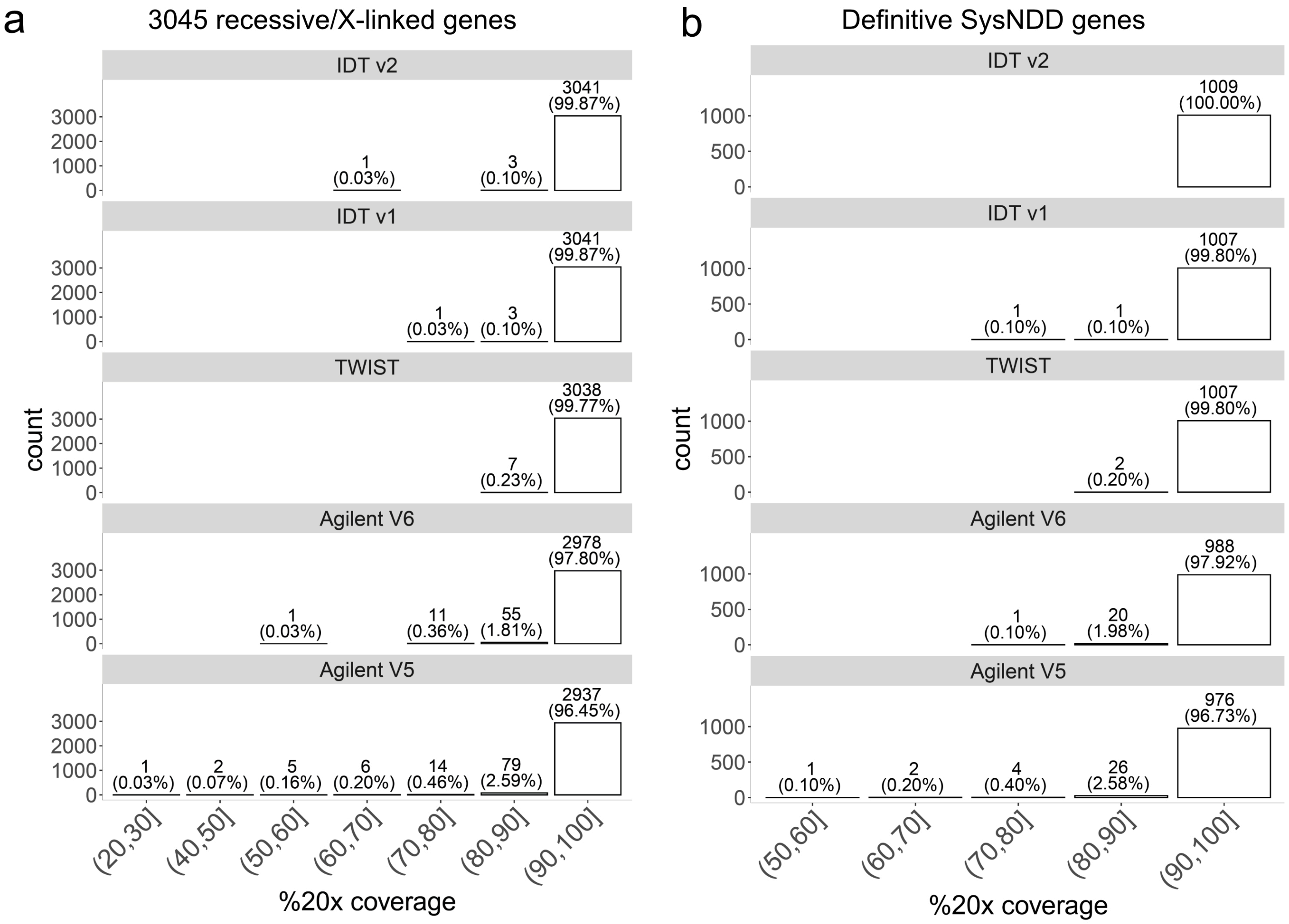

Supplementary Figure 1: 20x exome coverage of genes tested in our study.

Bar plots show number and percentage of targeted genes (count) with respective percentage of 20x coverage of their coding region (intervals of >20-30%, >30-40%, >40-50%, >50-60%, >60-70%, >70-80%, >80-90%, >90-100% 20x coverage of a gene), averaged from three representative samples each. Distribution of all investigated recessive/X-linked genes (**a**), as well as recessive/X-linked genes annotated in the SysNDD database as definitive genes for neurodevelopmental disorders which include developmental delay (DD), intellectual disability (ID) and autism spectrum disorder (ASD) (**b**), captured by the five exome capture kits (Agilent SureSelect XT Clinical Research Exome Kit (V5), or Human All Exon (V6), Twist Human Core Exome Kit (Twist Bioscience), and xGen Exome Research Panel (IDT v1.0 or IDT v2.0)). Numbers on bars indicate gene counts having the respective percentage of 20x coverage in each interval. Among the 700 test individuals, exome sequencing had been performed using Agilent V5 for 37 individuals, Agilent V6 for 348 individuals, TWIST for 16 individuals, IDT v1 for 70 individuals, and IDT v2 for 229 individuals. Data

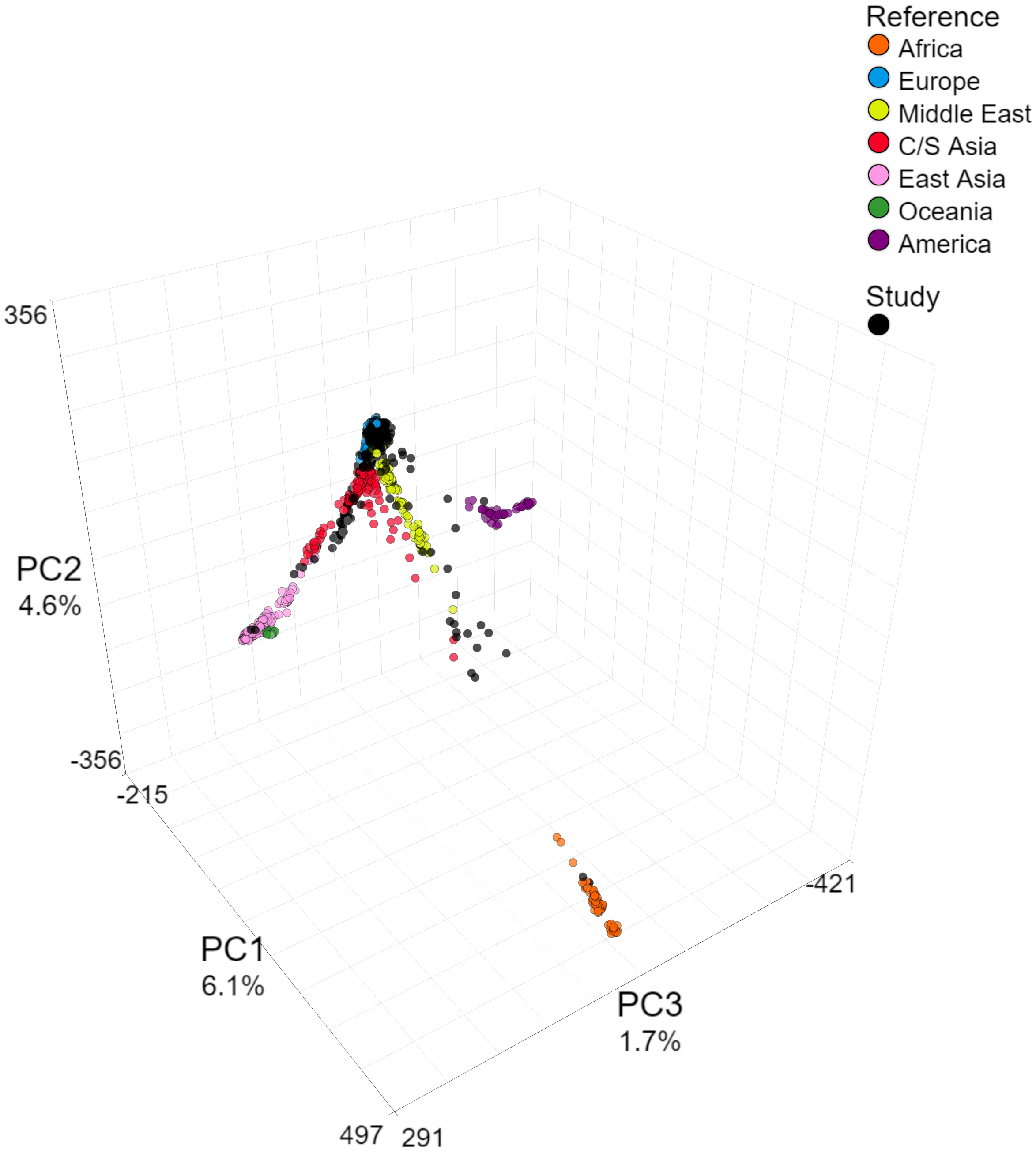

Supplementary Figure 2: Ancestry estimation by LASER (Locating Ancestry from SEquence Reads) analysis.

Dot plot shows clusters of our 700 individuals (black dots) among the main reference populations estimated by LASER (Locating Ancestry from SEquence Reads) analysis using sequence reads. Briefly, raw BAM files were converted to more compact LASER's native sequence format, and loci with less than 20 reads in more than 10% of individuals were removed, resulting in 16,039 loci. These loci were subjected to the LASER tool against the same loci from the reference populations from the Human Genome Diversity Project (HGDP), which were available in the LASER tool. The output file contained three principal components, which were used for clustering analysis by the k-nearest neighbors algorithm among the reference samples. The obtained knn clustering model using R package "class" showed an accuracy of 0.995 and an error rate of 0.005, which was then used to cluster the study samples.

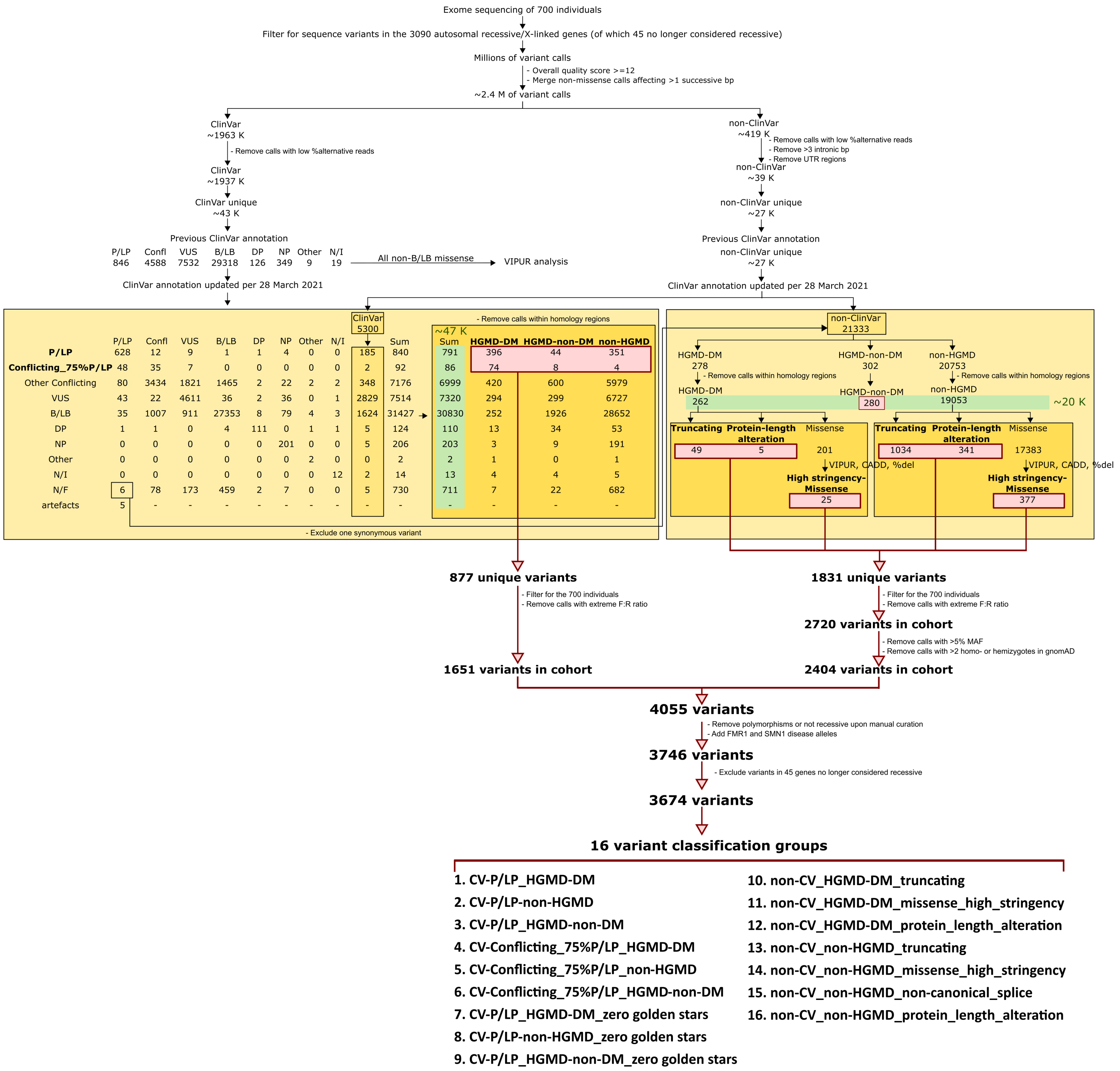
Exome sequencing of 700 individuals
Filter for sequence variants in the 3090 autosomal recessive/X-linked genes (of which 45 no longer considered recessive)
Millions of variant calls
- Overall quality score >=12
- Merge non-missense calls affecting >1 successive bp
~2.4 M of variant calls
ClinVar ~1963 K
- Remove calls with low %alternative reads
ClinVar ~1937 K
ClinVar unique ~43 K
Previous ClinVar annotation
P/LP 846 | Confl 4588 | VUS 7532 | B/LB 29318 | DP 126 | NP 349 | Other 9 | N/I 19
All non-B/LB missense
VIPUR analysis
ClinVar annotation updated per 28 March 2021
non-ClinVar ~419 K
- Remove calls with low %alternative reads
- Remove >3 intronic bp
- Remove UTR regions
non-ClinVar ~39 K
non-ClinVar unique ~27 K
Previous ClinVar annotation
non-ClinVar unique ~27 K
ClinVar annotation updated per 28 March 2021
ClinVar 5300
- Remove calls within homology regions
~47 K
	P/LP	Confl	VUS	B/LB	DP	NP	Other	N/I	ClinVar 5300	Sum	Sum	HGMD-DM	HGMD-non-DM	non-HGMD
P/LP	628	12	9	1	1	4	0	0	185	840	791	396	44	351
Conflicting_75%P/LP	48	35	7	0	0	0	0	0	2	92	86	74	8	4
Other Conflicting	80	3434	1821	1465	2	22	2	2	348	7176	6999	420	600	5979
VUS	43	22	4611	36	2	36	0	1	2829	7514	7320	294	299	6727
B/LB	35	1007	911	27353	8	79	4	3	1624	31427	30830	252	1926	28652
DP	1	1	0	4	111	0	1	1	5	124	110	13	34	53
NP	0	0	0	0	0	201	0	0	5	206	203	3	9	191
Other	0	0	0	0	0	0	2	0	0	2	2	1	0	1
N/I	0	0	0	0	0	0	0	12	2	14	13	4	4	5
N/F	6	78	173	459	2	7	0	0	5	730	711	7	22	682
artefacts	5	-	-	-	-	-	-	-	-	-	-	-	-	-
- Exclude one synonymous variant
non-ClinVar 21333
HGMD-DM 278
HGMD-non-DM 302
non-HGMD 20753
- Remove calls within homology regions
- Remove calls within homology regions
HGMD-DM 262
HGMD-non-DM 280
non-HGMD 19053
~20 K
Truncating | Protein-length alteration | Missense
49 | 5 | 201
VIPUR, CADD, %del
High stringency-Missense 25
Truncating | Protein-length alteration | Missense
1034 | 341 | 17383
VIPUR, CADD, %del
High stringency-Missense 377
877 unique variants
- Filter for the 700 individuals
- Remove calls with extreme F:R ratio
1651 variants in cohort
1831 unique variants
- Filter for the 700 individuals
- Remove calls with extreme F:R ratio
2720 variants in cohort
- Remove calls with >5% MAF
- Remove calls with >2 homo- or hemizygotes in gnomAD
2404 variants in cohort
4055 variants
- Remove polymorphisms or not recessive upon manual curation
- Add FMR1 and SMN1 disease alleles
3746 variants
- Exclude variants in 45 genes no longer considered recessive
3674 variants
16 variant classification groups
1. CV-P/LP_HGMD-DM
2. CV-P/LP-non-HGMD
3. CV-P/LP_HGMD-non-DM
4. CV-Conflicting_75%P/LP_HGMD-DM
5. CV-Conflicting_75%P/LP_non-HGMD
6. CV-Conflicting_75%P/LP_HGMD-non-DM
7. CV-P/LP_HGMD-DM_zero golden stars
8. CV-P/LP-non-HGMD_zero golden stars
9. CV-P/LP_HGMD-non-DM_zero golden stars
10. non-CV_HGMD-DM_truncating
11. non-CV_HGMD-DM_missense_high_stringency
12. non-CV_HGMD-DM_protein_length_alteration
13. non-CV_non-HGMD_truncating
14. non-CV_non-HGMD_missense_high_stringency
15. non-CV_non-HGMD_non-canonical_splice
16. non-CV_non-HGMD_protein_length_alteration

Supplementary Figure 3: Variant filtering.

700 healthy individuals (350 parental couples) were screened for variants in 3,090 recessive genes using exome sequencing data. Variants were filtered for an overall quality score ≥12. Non-missense variants affecting more than one successive bp were merged to one variant call. Possible artefacts observed in <28% alternative reads in the proband as well as in multiple probands with an average of <28% alternative reads in at least 90% of these probands were discarded. All variants were separated into variants annotated and not annotated in the ClinVar database. The non-ClinVar variants in the untranslated region, farther than three intronic bp, as well as synonymous non-ClinVar variants were excluded. Only different variants were now obtained for further steps. Non-B/LB ClinVar missense variants were subjected to VIPUR analysis for a benchmark dataset. Annotaions of all ClinVar variants were updated per 28 March 2021. Variants within homology regions were excluded and the remaining separated according to HGMD classifications. The non-ClinVar variants were divided into different functional classes, where missense variants were subjected to VIPUR analysis, and annotated with CADD and eight other prediction tools (SIFT, PolyPhen2, LRT, MutationTaster, MutationAccessor, FATHMM, PROVEAN, M.CAP) to evaluate "high stringency" missense variants using the benchmark dataset obtained from the ClinVar missense variants. The obtained variants were filtered for the 700 individuals. Likely artefacts, which exhibited an extreme forward-to-reverse read ratio (<0.2 or >0.8), were removed. The non-ClinVar variants with an overall minor allele frequency >5%, a cohort-specific allele frequency >5%, or with >2 homo- or hemizygotes reported in the gnomAD database were excluded. Variants in genes with carrier frequency >2% in our cohort were manually curated to exclude likely polymorphisms or dominant alleles before calculating the final carrier frequencies. After the addition of the *FMR1* and *SMN1* disease alleles and exclusion of variants in 45 genes that were no longer considered recessive, 3,674 variants (in 3,046 genes) were obtained and classified according to evidence levels of pathogenicity into 16 variant classification groups, for which the final carrier frequencies were calculated and used for the carrier analysis. %del percentage of deleterious predictions, B/LB (likely) benign, Confl conflicting, CV ClinVar, DM disease-causing mutation, DP disease polymorphism, HGMD the Human Gene Mutation Database, N/I no interpretation of the single variant, N/F not found, NP not provided, P/LP (likely) pathogenic, VUS variant of uncertain significance.

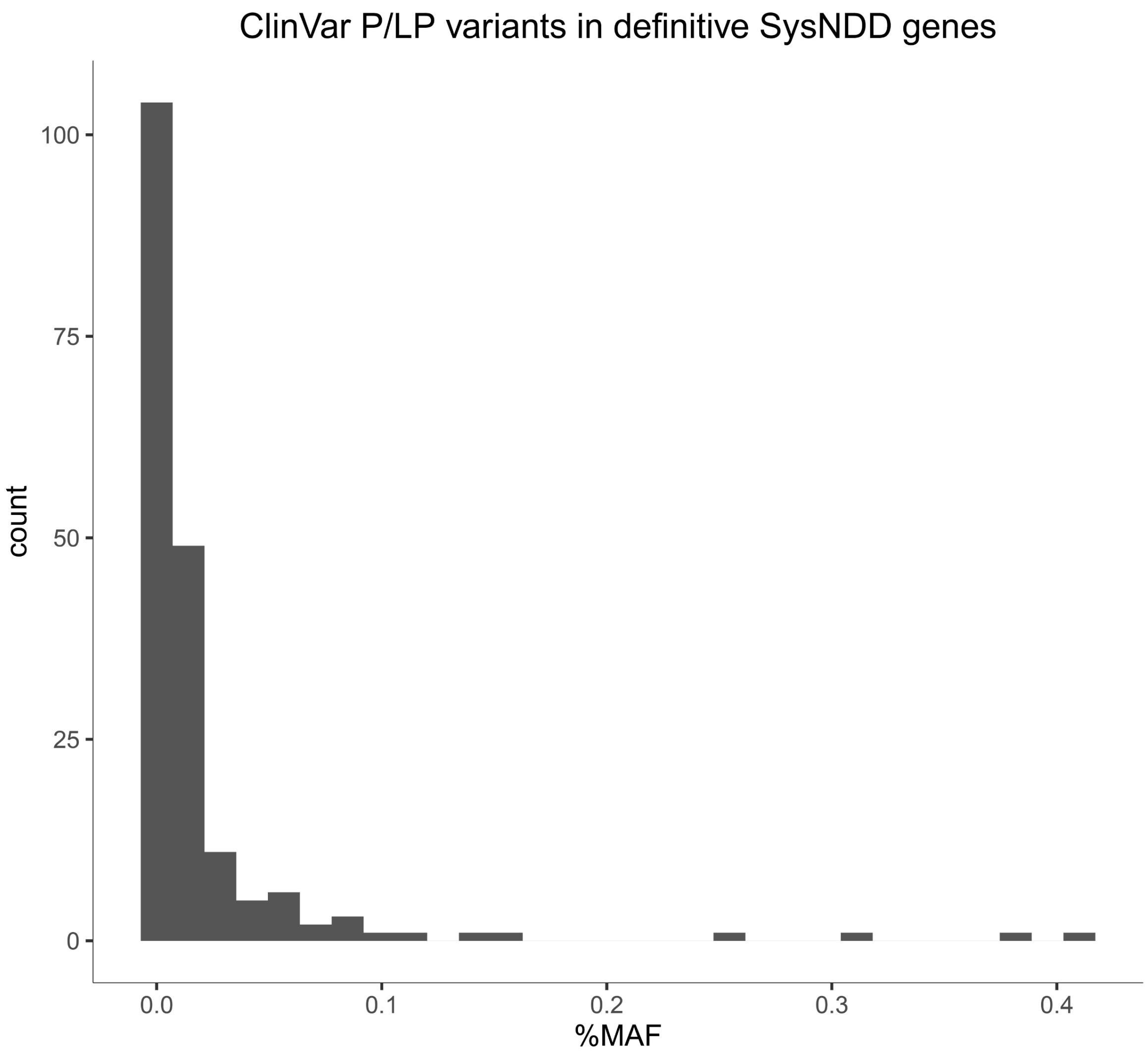

Supplementary Figure 4: Distribution of all population minor allele frequencies of ClinVar P/LP variants in SysNDD genes.

Histogram shows distribution of all population minor allele frequencies (MAFs) of pathogenic/likely pathogenic variants in genes annotated in the SysNDD database as definitive genes for neurodevelopmental disorders, which include developmental delay (DD), intellectual disability (ID) and autism spectrum disorder (ASD).

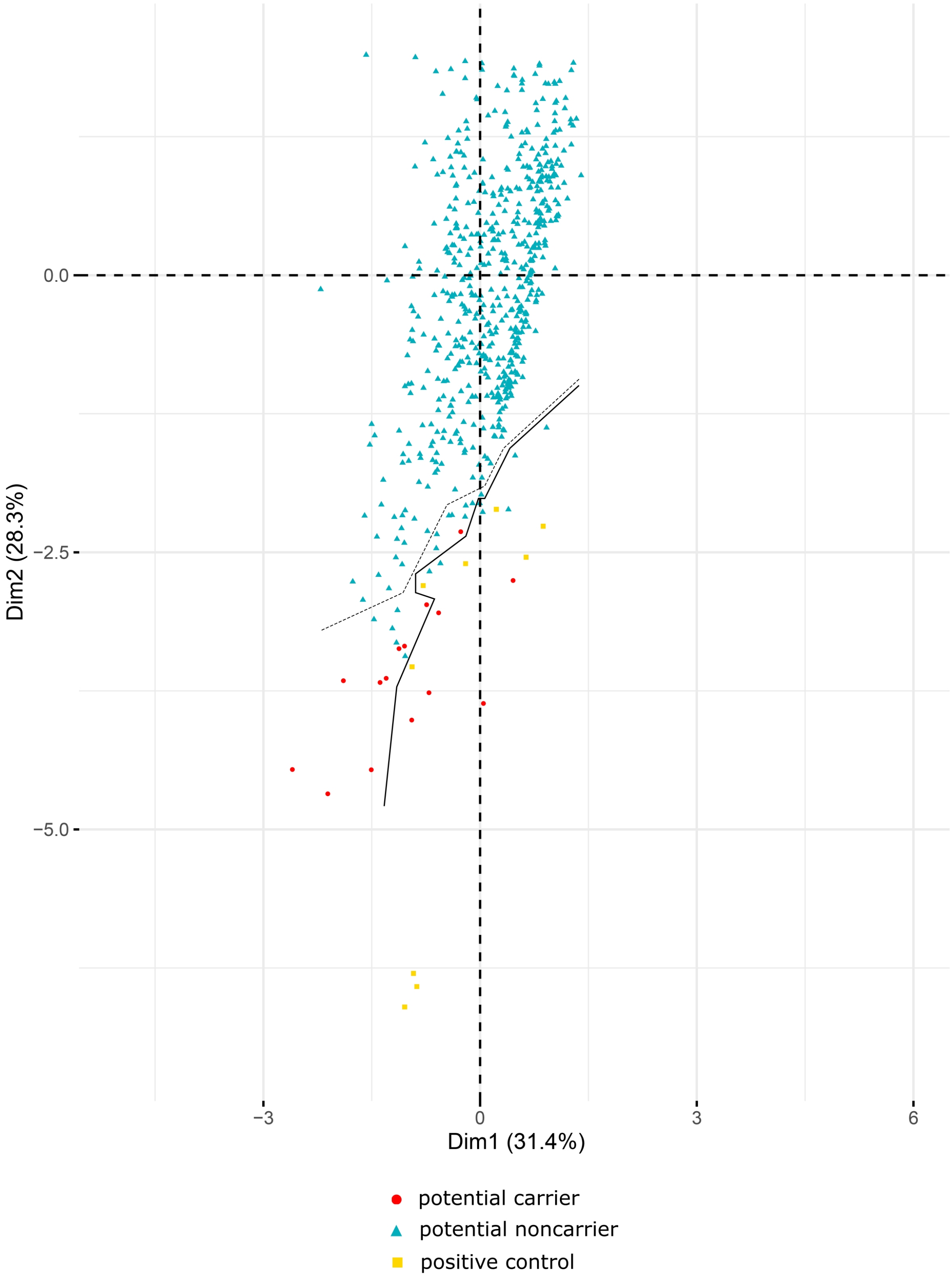

Supplementary Figure 5: Principal component analysis for the identification of potential *SMN1* carriers from exome sequencing data.

Dot plot shows clusters of potential carriers, noncarriers, and positive controls for the recurrent deletion of ex7/8 in the *SMN1* gene by principal component analysis (PCA) of the parameters obtained by calculation of the scale factor and the raw proportion of *SMN1*/*SMN2* reads at the three positions that are specific to *SMN1* according to a method based on exome sequencing data recently described by Lopez-Lopez, D. et al 2020. A dashed black line indicates a border used to separate potential carriers for testing with MLPA. The black line indicates a border that separates true positive carriers, confirmed by MLPA.

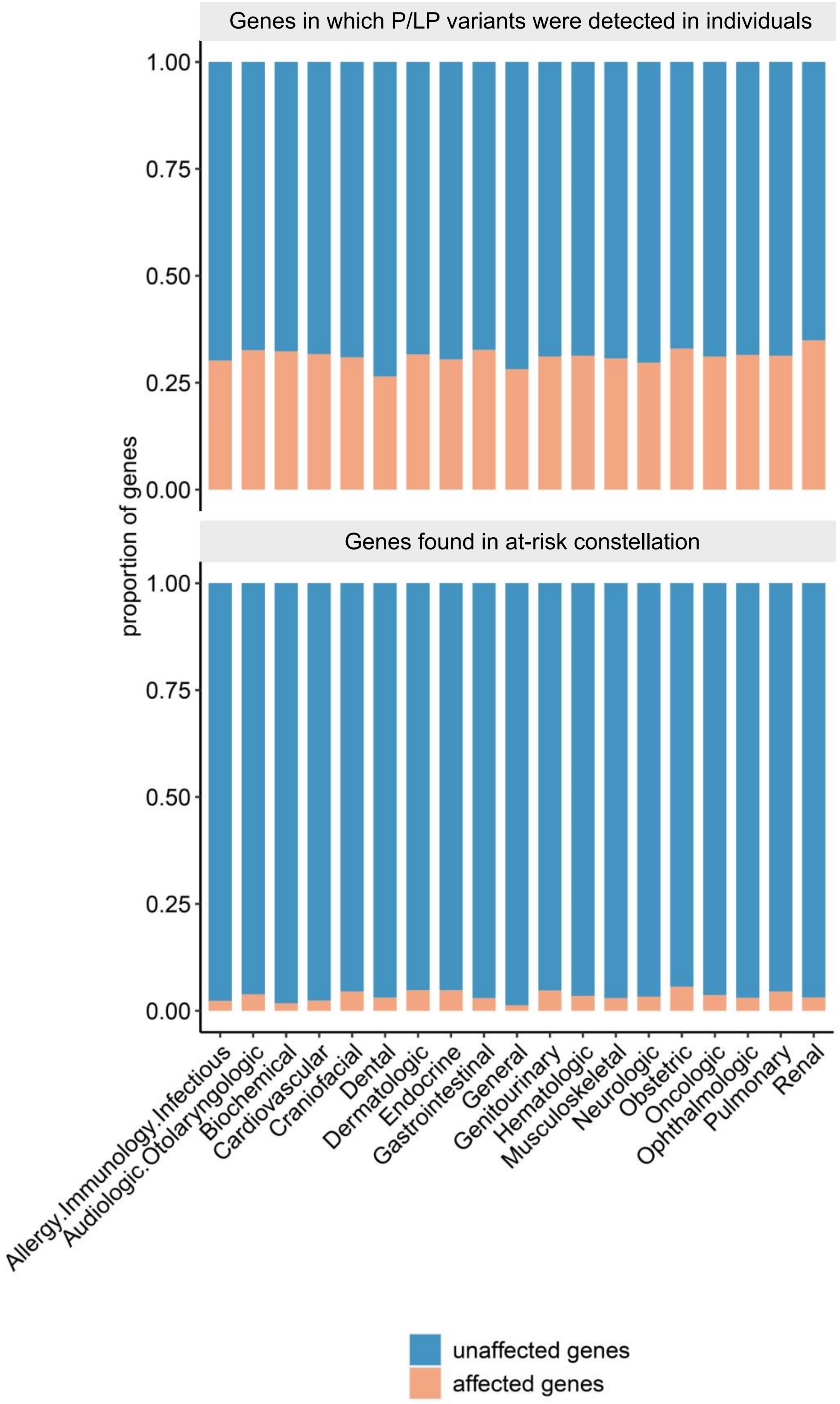

Supplementary Figure 6: Distribution of genes in which P/LP variants were detected with reference to manifestation categories.

Bar plots show distributions of the proportions of genes in which P/LP variants were detected in individuals (upper panel) and of genes in which P/LP variants were detected in both mother and father (at-risk constellation) (lower panel) considering the first 14 variant classification groups normalized to the total number of genes in each manifestation category. No category was significantly enriched. P/LP pathogenic/likely pathogenic.

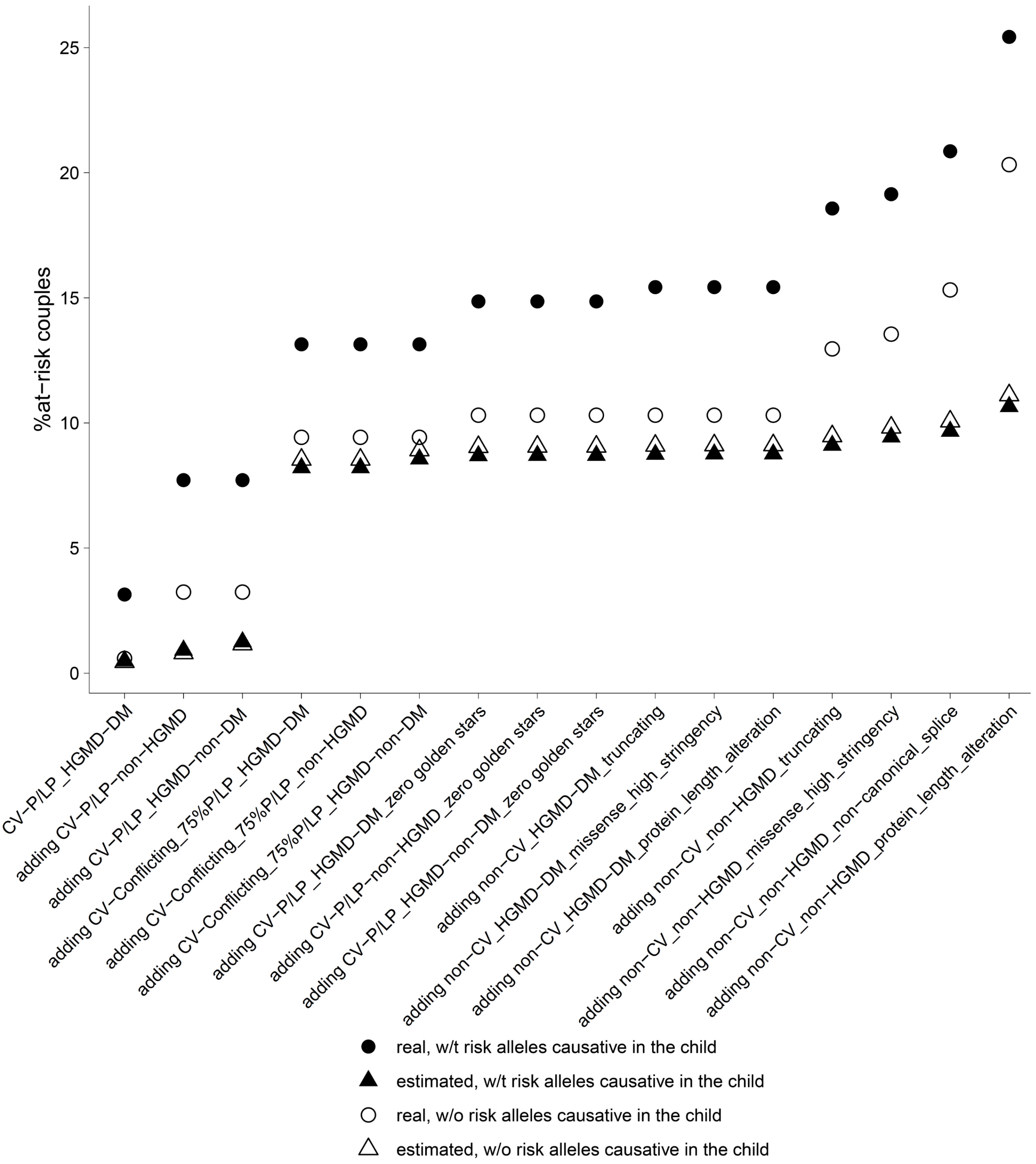

Supplementary Figure 7: Actual versus estimated at-risk couple frequencies.

Dot plots show the percentage of real versus estimated at-risk couple frequencies according to variant classification groups with or without at-risk alleles that were inherited in the diagnosed children.

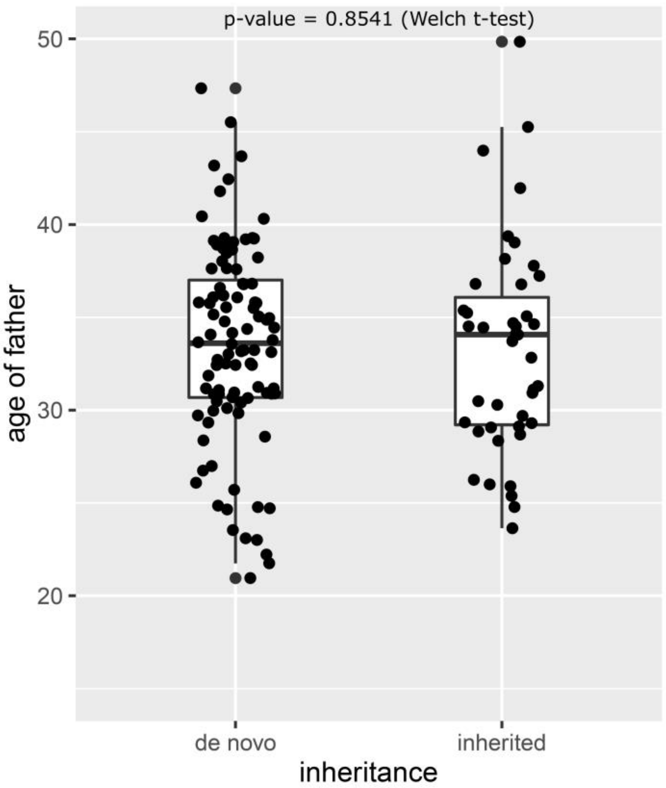

Supplementary Figure 8: Distribution of paternal age in children with causal *de novo* and inherited pathogenic variants, respectively.

Dot plot shows no effect of paternal age on the inheritance of causative alleles in the respectively affected children. Box plot shows median and interquatile range (IQR), the upper whisker indicates Q3 + 1.5 IQR,whereas the lower whisker Q1 – 1.5 IQR.

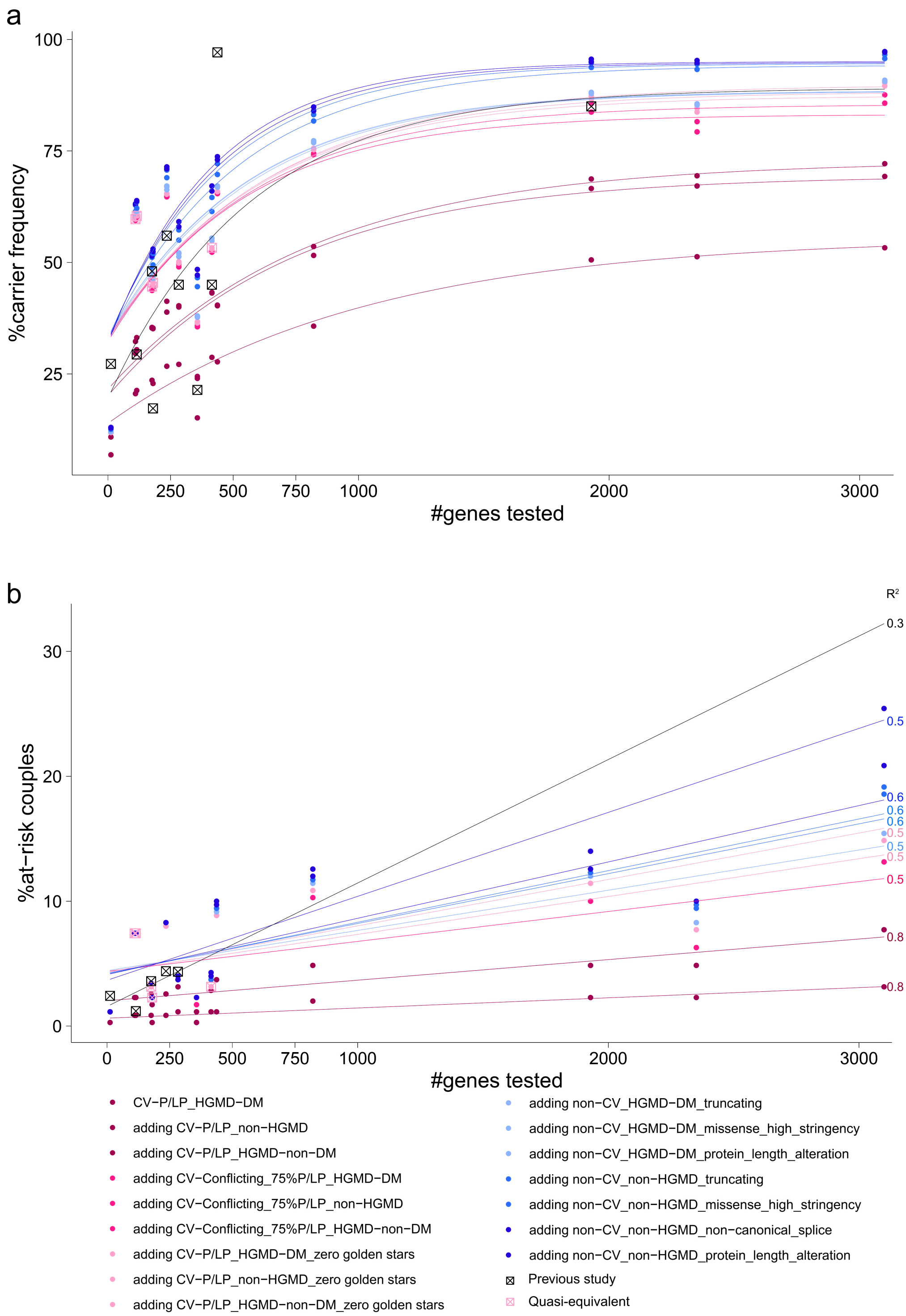

Supplementary Figure 9: Comparison of carrier and at-risk rates between carrier studies.

Percentage of carriers (**a**) and real at-risk couples (**b**) were compared according to variant classification groups (dark red to light yellow circle) between our study (adjusted to the respective gene numbers) and previous carrier screening studies (black circle), including Hernandez-Nieto C, et al Prenat. Diagn. 2020; Haque IS, et al JAMA 2016; Guo MH, et al Genet. Med. 2019; Peyser A, et al Genet Med 2019; Zhao S, et al Eur J Hum Genet 2019; Ben-Shachar R, et al Genet Med 2019; Abouelhoda M, et al Genet Med 2016; Sallevelt SCEH, et al Genet Med 2021; Bell CJ, et al Sci. Transl. Med 2011; Hogan GJ, et al Clin. Chem 2018; Fridman H, et al Genet Med 2020; Beauchamp KA et al, Genet Med 2019. Black crossed rectangle indicates values from previous studies wherever available. Non-black crossed rectangle indicates our respective variant classification group that was quasi-equivalent to the variant classifications reported in the respective studies only some of which stated P/LP definitions, which were mostly based on ACMG-guidelines. Hence, for these studies our variants including until CV-P/LP_HGMD-non-DM_zero golden stars (light pink) were considered quasi-equivalent for comparison. Most previous studies analyzed less than 500 genes, two studies ~1,900 and ~2,300 genes. To fill up the gap, we also simulated a union of all genes screened by all these previous studies, accounting for ~800 genes. R-squares by linear regression analysis were shown for the relationship between at-risk couple frequencies and numbers of genes tested.

Supplementary tables

Supplementary Table 2: Literature search for the genes with autosomal dominant inheritance in DDG2P and ClinGen.

Nr.	Approved gene symbol	Inheritance in CGD and ClinGen	Our literature search	References
1	*DSP*	AD	AR/AD	Favre B 2018; Jan A 2015; Marutahappu T 2019
2	*EXT2*	AD	AR/AD	El-Bazzal L 2019
3	*FGFR3*	AD	AR/AD	Yang K 2019
4	*MPZ*	AD	AR/AD	Al-Thihli et al. 2008; Takashima et al. 2002
5	*PAX3*	AD	AR/AD	Mousty et al., 2015; Pingault et al., 2010; Saberi et al., 2018
6	*RAD21*	AD	AR/AD	Bonora E 2015
7	*SCN1B*	AD	AR/AD	Ramadan W 2017
8	*SLC2A1*	AD	AR/AD	Wang et al., 2000; Klepper et al., 2009
9	*STIM1*	AD	AR/AD	Silva-Rojas R 2020

KEY: AD - autosomal dominant, AR - autosomal recessive

Supplementary Table 3a: Summary of carrier analysis results for autosomal recessive and X-linked genes according to increasing levels of variant pathogenicity thresholds.

Approach	#variants	#genes	#variants/individual	#genes/individual	#carriers	%carriers	#at-risk variants	#at-risk genes	#at-risk couples	%at-risk couples	#biparentally transmitted causative	%risk reduction potential	%estimated at-risk couples
CV-P/LP_HGMD-DM	317	249	1	1	370	52.9	14	10	11	3.1	7	2.0	0.5
adding CV-P/LP-non-HGMD	598	413	1	1	484	69.1	34	24	27	7.7	13	3.7	0.9
adding CV-P/LP_HGMD-non-DM	629	426	1	1	504	72.0	34	24	27	7.7	13	3.7	1.2
adding CV-Conflicting_75%P/LP_HGMD-DM	702	457	2	2	599	85.6	46	31	46	13.1	13	3.7	8.2
adding CV-Conflicting_75%P/LP_non-HGMD	705	460	2	2	599	85.6	46	31	46	13.1	13	3.7	8.2
adding CV-Conflicting_75%P/LP_HGMD-non-DM	712	465	2	2	612	87.4	46	31	46	13.1	13	3.7	8.5
adding CV-P/LP_HGMD-DM_zero golden stars	771	512	2	2	625	89.3	55	39	52	14.9	17	4.9	8.7
adding CV-P/LP-non-HGMD_zero golden stars	820	549	2	2	630	90.0	55	39	52	14.9	17	4.9	8.7
adding CV-P/LP_HGMD-non-DM_zero golden stars	821	550	2	2	630	90.0	55	39	52	14.9	17	4.9	8.7
adding non-CV_HGMD-DM_truncating	871	575	2	2	632	90.3	58	41	54	15.4	19	5.4	8.7
adding non-CV_HGMD-DM_missense_high_stringency	896	587	2	2	633	90.4	58	41	54	15.4	19	5.4	8.7
adding non-CV_HGMD-DM_protein_length_alteration	901	590	2	2	634	90.6	58	41	54	15.4	19	5.4	8.7
adding non-CV_non-HGMD_truncating	1606	1009	4	3	669	95.6	77	56	65	18.6	25	7.1	9.1
adding non-CV_non-HGMD_missense_high_stringency	1961	1168	4	4	675	96.4	82	61	67	19.1	27	7.7	9.4
adding non-CV_non-HGMD_non-canonical_splice	2175	1269	5	4.5	679	97.0	92	71	73	20.9	27	7.7	9.6
adding non-CV_non-HGMD_protein_length_alteration	2455	1367	5	5	681	97.3	107	81	87	24.9	27	7.7	10.5

KEY: CV - ClinVar, P/LP - pathogenic/likely pathogenic, HGMD - Human Gene Mutation Database, DM - disease mutation

Supplementary Table 3b: Summary of carrier analysis results for autosomal recessive genes according to increasing levels of variant pathogenicity thresholds.

Approach	#variants	#genes	#variants/individual	#genes/individual	#carriers	%carriers	#at-risk variants	#at-risk genes	#at-risk couples	%at-risk couples	#biparentally transmitted causative	%risk reduction potential	%estimated at-risk couples
CV-P/LP_HGMD-DM	315	247	1	1	367	52.4	12	8	8	2.3	5	1.4	0.5
adding CV-P/LP-non-HGMD	590	405	1	1	480	68.6	26	16	19	5.4	8	2.3	0.9
adding CV-P/LP_HGMD-non-DM	621	418	1	1	501	71.6	26	16	19	5.4	8	2.3	1.2
adding CV-Conflicting_75%P/LP_HGMD-DM	693	448	2	2	597	85.3	37	22	37	10.6	8	2.3	8.2
adding CV-Conflicting_75%P/LP_non-HGMD	696	451	2	2	597	85.3	37	22	37	10.6	8	2.3	8.2
adding CV-Conflicting_75%P/LP_HGMD-non-DM	703	456	2	2	610	87.1	37	22	37	10.6	8	2.3	8.5
adding CV-P/LP_HGMD-DM_zero golden stars	762	503	2	2	624	89.1	46	30	43	12.3	12	3.4	8.7
adding CV-P/LP-non-HGMD_zero golden stars	811	540	2	2	629	89.9	46	30	43	12.3	12	3.4	8.7
adding CV-P/LP_HGMD-non-DM_zero golden stars	812	541	2	2	629	89.9	46	30	43	12.3	12	3.4	8.7
adding non-CV_HGMD-DM_truncating	862	566	2	2	631	90.1	49	32	45	12.9	14	4.0	8.7
adding non-CV_HGMD-DM_missense_high_stringency	887	578	2	2	632	90.3	49	32	45	12.9	14	4.0	8.7
adding non-CV_HGMD-DM_protein_length_alteration	892	581	2	2	633	90.4	49	32	45	12.9	14	4.0	8.7
adding non-CV_non-HGMD_truncating	1586	990	3	3	669	95.6	59	39	51	14.6	18	5.1	9.1
adding non-CV_non-HGMD_missense_high_stringency	1940	1148	4	4	675	96.4	63	43	52	14.9	19	5.4	9.4
adding non-CV_non-HGMD_non-canonical_splice	2147	1242	4.5	4	679	97.0	67	47	54	15.4	19	5.4	9.6
adding non-CV_non-HGMD_protein_length_alteration	2421	1336	5	5	680	97.1	79	55	62	17.7	19	5.4	10.5

KEY: CV - ClinVar, P/LP - pathogenic/likely pathogenic, HGMD - Human Gene Mutation Database, DM - disease mutation

Supplementary Table 3c: Summary of carrier analysis results for X-linked genes according to increasing levels of variant pathogenicity thresholds.

Approach	#variants*	#genes	#variants/individual	#genes/individual	#carriers	%carriers	#at-risk variants	#at-risk genes	#at-risk couples	%at-risk couples	#biparentally transmitted causative	%risk reduction potential	%estimated at-risk couples
CV-P/LP_HGMD-DM	2	2	0	0	3	0.4	2	2	3	0.9	2	0.6	0.0008
adding CV-P/LP-non-HGMD	8	8	0	0	9	1.3	8	8	9	2.6	5	1.4	0.0008
adding CV-Conflicting_75%P/LP_HGMD-DM	9	9	0	0	11	1.6	9	9	11	3.1	5	1.4	0.0016
adding non-CV_non-HGMD_truncating	20	19	0	0	21	3.0	18	17	19	5.4	7	2.0	0.0025
adding non-CV_non-HGMD_missense_high_stringency	21	20	0	0	22	3.1	19	18	20	5.7	8	2.3	0.0025
adding non-CV_non-HGMD_non-canonical_splice	28	27	0	0	29	4.1	25	24	26	7.4	8	2.3	0.0025
adding non-CV_non-HGMD_protein_length_alteration	34	30	0	0	40	5.7	28	26	33	9.4	8	2.3	0.0180

KEY: CV - ClinVar, P/LP - pathogenic/likely pathogenic, HGMD - Human Gene Mutation Database, DM - disease mutation

*No X-linked variants with zero golden stars

Supplementary Table 6: Couples with recessive genetic diagnoses in their affected child not passing the stringent filtering criteria in carrier-screening.

Couple ID	Approved gene symbol	Variants	Variants in genomic position	Reason
027	*PTPN23*	NM_015466.3:c.[2878_2889del];[3970C>T] p.[(Gln960_Pro963del)];[(Arg1324Cys)]	hg19 chr3:g.[47452166_47452177del];[47453354C>T]	missense not high stringency VIPUR: 0.6033; CADD: 33.0; %del: 75
039	*DOCK8*	NM_203447.3:c.[314C>G];[3023G>A] p.[(Pro105Arg)];[(Arg1008Gln)]	hg19 chr9:g.[286618C>G];[396837G>A]	both VUS in ClinVar
045	*KDM5C*	NM_004187.3:c.[2212T>C];[0] p.[(Cys738Arg)];[0]	hg19 chrX:g.[53228190T>C];[0]	missense not high stringency VIPUR: 0.791; CADD: 23.0; %del: 100
064	*DHTKD1*	NM_018706.5:c.[2185G>A];[2185G>A] p.[(Gly729Arg)];[(Gly729Arg)]	hg19 chr10:g.[12154929G>A];[12154929G>A]	variant with conflicting interpretations in ClinVar with <75% P/LP entries
151	*SHROOM4*	NM_020717.3:c.[4394A>G];[0] p.[(Gln1465Arg)];[0]	hg19 chrX:g.[50339783A>G];[0]	missense not high stringency VIPUR: 0.5644; CADD: 25.3; %del: 87.5
161	*RAB39B*	NM_171998.3:c.[29G>A];[0] p.[(Arg10Gln)];[0]	hg19 chrX:g.[154493545G>A];[0]	missense not high stringency VIPUR: 0.5581; CADD: 33.0; %del: 62.5
196	*ACO2*	NM_001098.2:c.[1859G>A];[2048G>T] p.[(Gly620Asp)];[(Gly683Val)]	hg19 chr22:g.[41922363G>A];[41923386G>T]	missense Gly683Val not high stringency VIPUR: 0.9284; CADD: 23.5; %del: 50
215	*COQ4*	NM_016035.3:c.[437T>G];[437T>G] p.[(Phe146Cys)];[(Phe146Cys)]	hg19 chr9:g.[131094466T>G];[131094466T>G]	ratio forward:reverse reads <20%
240	*BGN*	NM_001711.4:c.[855C>G];[0] p.[(Asp285Glu)];[0]	hg19 chrX:g.[152772589C>G];[0]	missense not high stringency VIPUR: 0.3464; CADD: 11.87; %del: 25
253	*BRWD3*	NM_153252.4:c.[5357G>A];[0] p.[(Arg1786His)];[0]	hg19 chrX:g.[79932160G>A];[0]	missense not high stringency VIPUR: n/a; CADD: 25.0; %del: 62.5
255	*MED12*	NM_005120.2:c.[2312T>C];[0] p.[(Ile771Thr)];[0]	hg19 chrX:g.[70345286T>C];[0]	missense not high stringency VIPUR: n/a; CADD: 24.7; %del: 75
256	*PLK4*	NM_014264.4:c.[1111C>T];[881T>G] p.[(Arg371*)];[(Ile294Ser)]	hg19 chr4:g.[128807636C>T];[128807406T>G]	missense not high stringency VIPUR: 0.0615; CADD: 7.855; %del: 0
260	*PLPBP*	NM_007198.3:c.[119C>T];[722G>A] p.[(Pro40Leu)];[(Arg241Gln)]	hg19 chr8:g.[37623063C>T];[37635516G>A]	missense Pro40Leu not high stringency VIPUR: 0.6855; CADD: 34.0; %del: 87.5

275	*PLPBP*	NM_007198.3:c.[249_252delGTCT];[614G>A]<br>p.[(Ser84Cysfs*21)];[(Arg205Gln)]	hg19 chr8:g.[37623800_37623804del];[37633452G>A]	missense not high stringency<br>VIPUR: 0.3709; CADD: 24.3; %del: 37.5
290	*PLK4*	NM_014264.4:c.[212C>T];[760_761insAC]<br>p.[(Ser71Phe)];[(Leu254Tyrfs*2)]	hg19 chr4:g.[128804502C>T];[128807286_128807287insAC]	missense not high stringency<br>VIPUR: 0.7322; CADD: 31.0; %del: 75
309	*SURF1*	NM_003172.3:c.[311C>T];[311C>T]<br>p.[(Pro104Leu)];[(Pro104Leu)]	hg19 chr9:g.[136221526G>A];[136221526G>A]	missense not high stringency<br>VIPUR: n/a; CADD: 24.5; %del: 75
